# Inhibition of extracellular vesicle‐derived miR‐146a‐5p decreases progression of melanoma brain metastasis via Notch pathway dysregulation in astrocytes

**DOI:** 10.1002/jev2.12363

**Published:** 2023-09-27

**Authors:** Emma Rigg, Jiwei Wang, Zhiwei Xue, Taral R. Lunavat, Guowei Liu, Tuyen Hoang, Himalaya Parajuli, Mingzhi Han, Rolf Bjerkvig, Petr V. Nazarov, Nathalie Nicot, Stephanie Kreis, Christiane Margue, Miléne Tetsi Nomigni, Jochen Utikal, Hrvoje Miletic, Terje Sundstrøm, Lars A. R. Ystaas, Xingang Li, Frits Thorsen

**Affiliations:** ^1^ Department of Neurosurgery, Qilu Hospital of Shandong University and Institute of Brain and Brain‐Inspired Science, Cheeloo College of Medicine Shandong University Jinan China; ^2^ Department of Biomedicine University of Bergen Bergen Norway; ^3^ Shandong Key Laboratory of Brain Function Remodeling Jinan China; ^4^ Department of Neurology, Molecular Neurogenetics Unit‐West, Massachusetts General Hospital Harvard Medical School Charlestown Massachusetts USA; ^5^ Bioinformatics Platform and Multiomics Data Science Research Group, Department of Cancer Research Luxembourg Institute of Health Luxembourg; ^6^ LuxGen Genome Center, Luxembourg Institute of Health Laboratoire National de Santé Dudelange Luxembourg; ^7^ Department of Life Sciences and Medicine University of Luxembourg Luxembourg; ^8^ Skin Cancer Unit German Cancer Research Center (DKFZ) Heidelberg Germany; ^9^ Department of Dermatology, Venereology and Allergology University Medical Center Mannheim, Ruprecht‐Karl University of Heidelberg Mannheim Germany; ^10^ DKFZ Hector Cancer Institute at the University Medical Center Mannheim Mannheim Germany; ^11^ Department of Pathology Haukeland University Hospital Bergen Norway; ^12^ Department of Neurosurgery Haukeland University Hospital Bergen Norway; ^13^ Department of Clinical Medicine University of Bergen Bergen Norway; ^14^ Molecular Imaging Center, Department of Biomedicine University of Bergen Bergen Norway

**Keywords:** brain metastasis, deserpidine, extracellular vesicles, melanoma, miR‐146a‐5p, normal human astrocytes (NHA)

## Abstract

Melanoma has the highest propensity of all cancers to metastasize to the brain with a large percentage of late‐stage patients developing metastases in the central nervous system (CNS). It is well known that metastasis establishment, cell survival, and progression are affected by tumour‐host cell interactions where changes in the host cellular compartments likely play an important role. In this context, miRNAs transferred by tumour derived extracellular vesicles (EVs) have previously been shown to create a favourable tumour microenvironment. Here, we show that miR‐146a‐5p is highly expressed in human melanoma brain metastasis (MBM) EVs, both in MBM cell lines as well as in biopsies, thereby modulating the brain metastatic niche. Mechanistically, miR‐146a‐5p was transferred to astrocytes via EV delivery and inhibited NUMB in the Notch signalling pathway. This resulted in activation of tumour‐promoting cytokines (IL‐6, IL‐8, MCP‐1 and CXCL1). Brain metastases were significantly reduced following miR‐146a‐5p knockdown. Corroborating these findings, miR‐146a‐5p inhibition led to a reduction of IL‐6, IL‐8, MCP‐1 and CXCL1 in astrocytes. Following molecular docking analysis, deserpidine was identified as a functional miR‐146a‐5p inhibitor, both in vitro and in vivo. Our results highlight the pro‐metastatic function of miR‐146a‐5p in EVs and identifies deserpidine for targeted adjuvant treatment.

## 1 INTRODUCTION

The incidence of cutaneous melanoma is increasing world‐wide where up to 60% of the patients with advanced disease will eventually be diagnosed with brain metastasis (Rishi & Yu, 2020). These patients often develop multiple metastases and even with modern therapies, the average survival is still quantified in months following diagnosis (Anvari et al., 2021). Treatment of melanoma brain metastasis (MBM) has proven to be a formidable clinical challenge. Tumours consistently acquire resistance to targeted therapies and immune checkpoint inhibitors. While having a good intracranial response in asymptomatic MBM patients, minimal effectiveness is seen in patients who are symptomatic (Boire et al., 2020; Larkin et al., 2019; Rishi & Yu, 2020; Tawbi et al., 2019). Given the role of putative tumour‐host cell interactions, investigation into the MBM microenvironment is necessary to understand why current treatments are not working, and for the development of new and effective clinical treatment strategies.

The brain microenvironment (BME) consists of neurons and glial cells, in addition to extracellular matrix components. In brain metastasis, molecular interactions between tumour cells and the normal brain forms the brain metastatic niche. During niche establishment, the brain changes from being a hostile environment to one that promotes metastasis formation and growth.

Astrocytes are the most abundant cell type in the central nervous system (CNS), where they exert vital roles in glial‐neuronal homeostasis and blood‐brain barrier (BBB) maintenance (Cacho‐Diaz et al., 2020). In the case of metastasis, astrocytes can be regarded as the first cell type that the extravasated tumour cells encounter, forming an activated phenotype that may promote MBM growth. This includes altered cytokine release, translational and transcriptional remodelling, as well as increased proliferative capacities (Placone et al., 2016). Thus, activated astrocytes have been found to surround brain metastases where they most likely contribute to tumour progression (Wasilewski et al., 2017).

A major contributing factor to niche remodelling is the transfer of small extracellular vesicles (EVs) of endocytic origin from tumour cells (Peinado et al., 2017; Tamas et al., 2022). The EVs shuttle proteins, DNA, and RNA, mostly in the form of miRNAs, to both local and distant cells (Cesi et al., 2016; Valadi et al., 2007). In brain metastasis, EV cargo has been found to both break down the BBB and alter the vascular environment to promote establishment of a secondary tumour (Rodrigues et al., 2019; Tominaga et al., 2015). It has been shown that EVs‐delivered miRNAs from primary tumour cells can have important role in tumorigenesis, angiogenesis, metastasis, and drug resistance (Bartel, 2018; Cesi et al., 2018). However, their targets, transcriptional networks and direct role in BM development are unclear (Hanniford et al., 2015). It is known that miRNAs regulate 30% of the human genes and a majority of these have been found to be tumour‐associated (Si et al., 2019). Therefore, a further understanding on how MBM EVs contributes to MBM niche establishment is warranted, as the mechanisms related to metastasis remain largely unknown.

Here, we show that MBM‐EVs play a major role in the progression of MBM through upregulation of miR‐146a‐5p. We identified the mRNA *NUMB* as a direct binding partner of miR‐146a‐5p. This is important since Numb is an inhibitor protein in the Notch signalling pathway, known to be implicated in melanoma pathogenesis (Hristova et al., 2021). Therefore, our findings strongly suggest that miR‐146a‐5p is important in MBM development and thus represents a therapeutic target. We further identified, through molecular docking analysis, deserpidine, an antipsychotic and antihypertensive drug, to be a functional miR‐146a‐5p inhibitor, both in vitro and in vivo.

## 2 MATERIALS AND METHODS

### 2.1 Cell lines and cell culture conditions

Written consent was obtained from the patients before tumour material was collected and subsequently used to prepare cell lines. The Regional Ethical Committee (REC) approved tissue collection, biobank storage of tumour biopsies, as well as development and use of cell lines (REC Approvals 2013/720 and 2020/65185). Cell line authentication was verified by short tandem repeat (STR) fingerprinting and the cells were regularly tested for mycoplasma. The H1, H2, H3 and H10 cell lines were established in our laboratory from patient biopsies of human MBM. The BRAF mutation status of the H1, H2, H3 and H10 cell lines was investigated by performing massive parallel sequencing of the tumour DNA, according to published protocols (Bischof et al., 2019). The H1, H2 and H10 cell lines are BRAF^V600E^ mutated, while the H3 cells are BRAF^L577F^ mutated. Normal human astrocytes (NHA) were purchased from ABM (Applied Biological Materials, Vancouver, Canada) and human melanocytes were obtained from ATCC (American Type Culture Collection, Manassas, VA, USA).

The H1 cells were transduced with two lentiviral vectors, encoding Dendra (a green fluorescent protein (GFP) variant) and luciferase to obtain the H1_DL2 cell line. Flow cytometric isolation of cells by GFP expression was performed (BD FACS Aria, Becton Dickinson, Franklin Lakes, NJ, USA). All cell lines were grown in Dulbecco's Modified Eagle′s medium (DMEM; Sigma‐Aldrich Inc., St. Louis, MO, USA), supplemented with 10% heat‐inactivated new‐born calf serum (Thermo Fischer Scientific, Waltham, MA, USA), 5 mg/mL plasmocin (Invivogen, Toulouse, France), 2% L‐glutamine (BioWhittaker, Verviers, Belgium), penicillin (100 IU/mL) and streptomycin (100 mL/mL) (BioWhittaker). The cells were cultured in a standard tissue incubator at 37°C, 100% humidity and 5% CO_2_, and trypsinated once they attained 75% confluency using 0.25% Trypsin/EDTA (BioWhittaker).

### 2.2 EV isolation and characterization

All EVs were isolated and characterized according to MISEV 2018 guidelines (Théry et al., 2018). The H1, H2, H3, NHA and melanocyte cell lines were seeded in **eight** T175 flasks per isolation in DMEM growth medium as described above, supplemented with EV‐depleted fetal bovine serum (FBS). FBS was depleted of EVs by centrifuging at 120,000 *g* for 18 h at 4°C. Conditioned medium (CM) was collected by pipetting after 48 h, and cells were reseeded for further EV production. The isolation of EVs was performed as previously described (Lunavat et al., 2017). Briefly, CM was centrifuged at 300 *g* for 5 min to remove cell debris. The collected supernatant was centrifuged again at 2000 *g* for 20 min at 4°C to remove apoptotic bodies, and then transferred to ultracentrifuge tubes and centrifuged at 16,500 *g* for 20 min to remove microvesicles. Finally, the supernatant was centrifuged at 120,000 *g* for 2 h to pellet exosomes termed as EVs and the supernatant was discarded. The remaining EV pellet was resuspended in 100–500 mL sterile‐filtered PBS and frozen at −80°C until further use.

For particle size determination, EVs were diluted with filtered PBS at 1:100−1:1000. The sizes and relative intensities of EVs were quantified using a Zetasizer Nano ZS (Malvern Panalytical, Worcestershire, UK) or a Multiple–Laser ZetaView® f‐NTA Nanoparticle Tracking Analyzer (Particle Metrix GmbH, Inning am Ammersee, Germany).

To visualize the cup‐shaped morphology and the membrane structure of isolated EVs, transmission electron microscopy (TEM) was used. Approximately 10 μg of EVs were mounted on formvar carbon coated copper grids and post‐fixed with 2.5% glutaraldehyde, followed by staining with 2% of uranyl acetate. Grids were dried and imaged using a Hitachi HT7800 transmission electron microscope (Hitachi High‐Tech Corporation, Tokyo, Japan).

Other materials and methods used in this study are provided in the Supplementary materials.

## 3 RESULTS

### 3.1 EVs penetrate the mouse brain and increase brain metastatic growth

**FIGURE 1:**
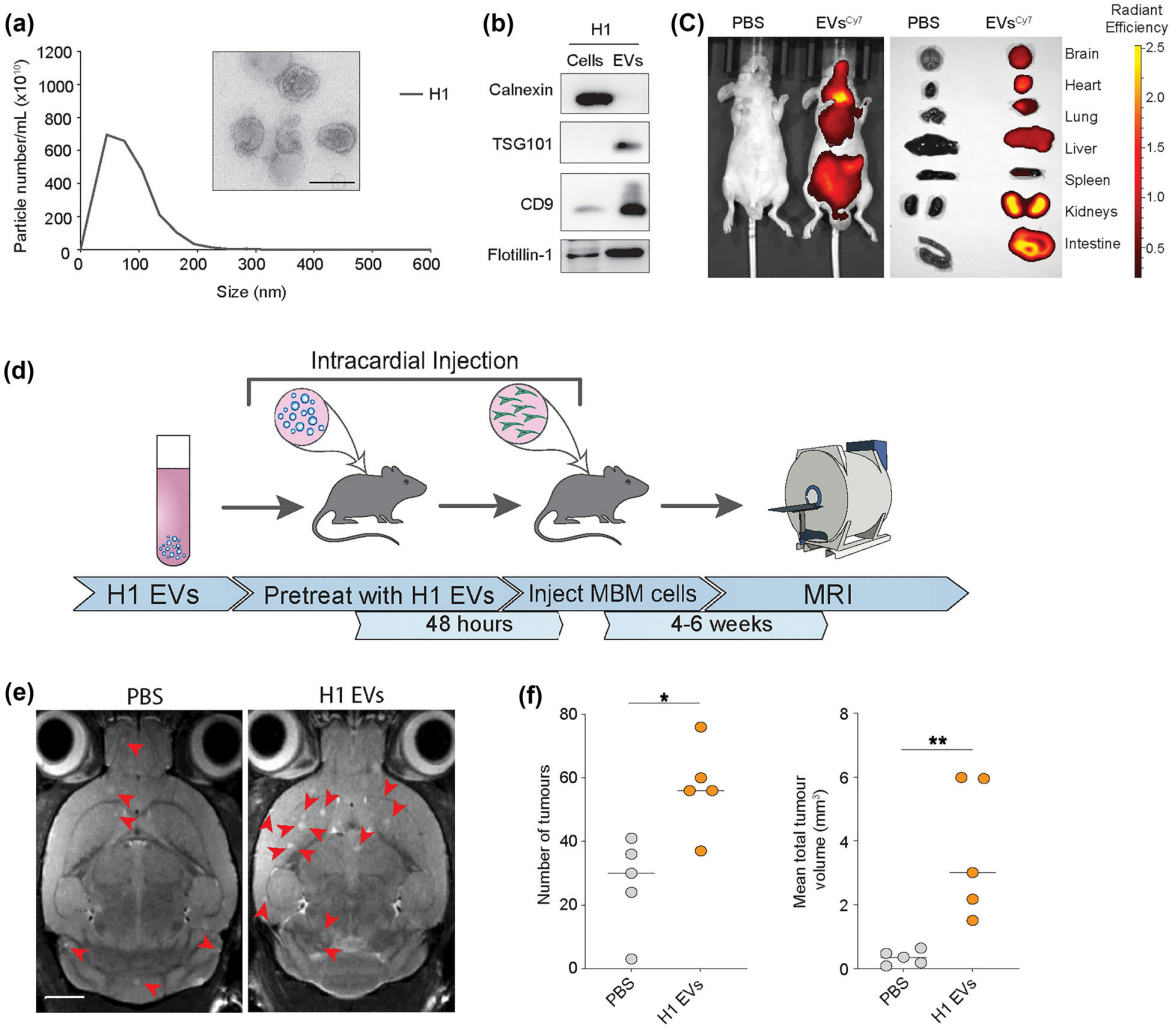
MBM‐EVs contribute to increased metastatic burden. (a) Representative nanoparticle tracking analysis using Malvern Nanosight and transmission electron microscopy of H1‐EVs. Size range 50–200 nm. Scale bar = 100 nm. (b) Western blot analysis of EV‐characteristic markers on H1 cells and corresponding EVs. (c) In vivo and ex vivo NIR imaging of mice and harvested major organs after injection of Cy7 (excitation/emission: 745/820 nm) H1‐EVs or PBS control. (d) Schematic workflow of the exosome‐primed in vivo metastatic model. (e) Development of brain metastasis assessed by T2 weighted MRI at week 4 after priming with MBM‐derived EVs or PBS prior to intracardial injection of MBM H1_DL2 cells. Scale bar = 2 mm. (f) Quantification of the total number and volume of brain metastasis at week 4 in exosome‐primed animals compared to the control PBS group. n.s. = not significant, *p < 0.05, **p < 0.01.

We first isolated EVs from two human MBM cell lines (H1, H2) as well as from normal human astrocytes (NHA) and melanocytes. Nanoparticle tracking revealed a mean EV diameter and concentration between 80 and 200 nm from NHA, H1, H2 and melanocytes (Figure 1a, Figure S1a), and the lipid bilayer membranes were verified by **TEM**(Figure 1a, Figure S1b). Additionally, Western blot (WB) analysis showed that the standard EV markers TSG101, CD9 and flotillin‐1 were positive, whereas the ER marker calnexin, which is known to be absent in EVs (Zhao et al., 2022), was negative (Figure 1b, Figure S1c). These data thus confirm the isolation of EVs.

To study whether tumour MBM‐EVs were taken up by the brain parenchyma in vivo, Cy7 labelled H1‐EVs were injected intracardially (i.c.) into the left cardiac ventricle of one female nude mouse. After 24 h, fluorescence imaging both in vivo and in organs ex vivo showed Cy7‐labelled EVs to be incorporated into major organs including the brain (Figure 1c). To assess the role of the MBM‐EVs in brain metastatic niche development, 5.0 × 10^9^ H1‐EVs were injected i.c into female NOD/SCID mice at 24 h intervals for 3 days (Figure 1d). 48 h after the final EV injection, 5.0 × 10^5^ H1_DL2 cells were injected i.c. and MBM development was monitored by magnetic resonance imaging (MRI; Figure 1e). Mice pretreated with H1‐EVs showed a significantly higher number of brain metastatic lesions at week 4 as well as significantly larger tumour volumes, compared to the control mice who received PBS pretreatment (Figure 1f).

These data show that circulating EVs from human MBM cells creates a brain metastatic niche that favours both brain metastatic initiation (number of brain metastases observed) as well as tumour growth.

### 3.2 EVs derived from human MBM cells activate NHAs and elevate cytokine production

**FIGURE 2:**
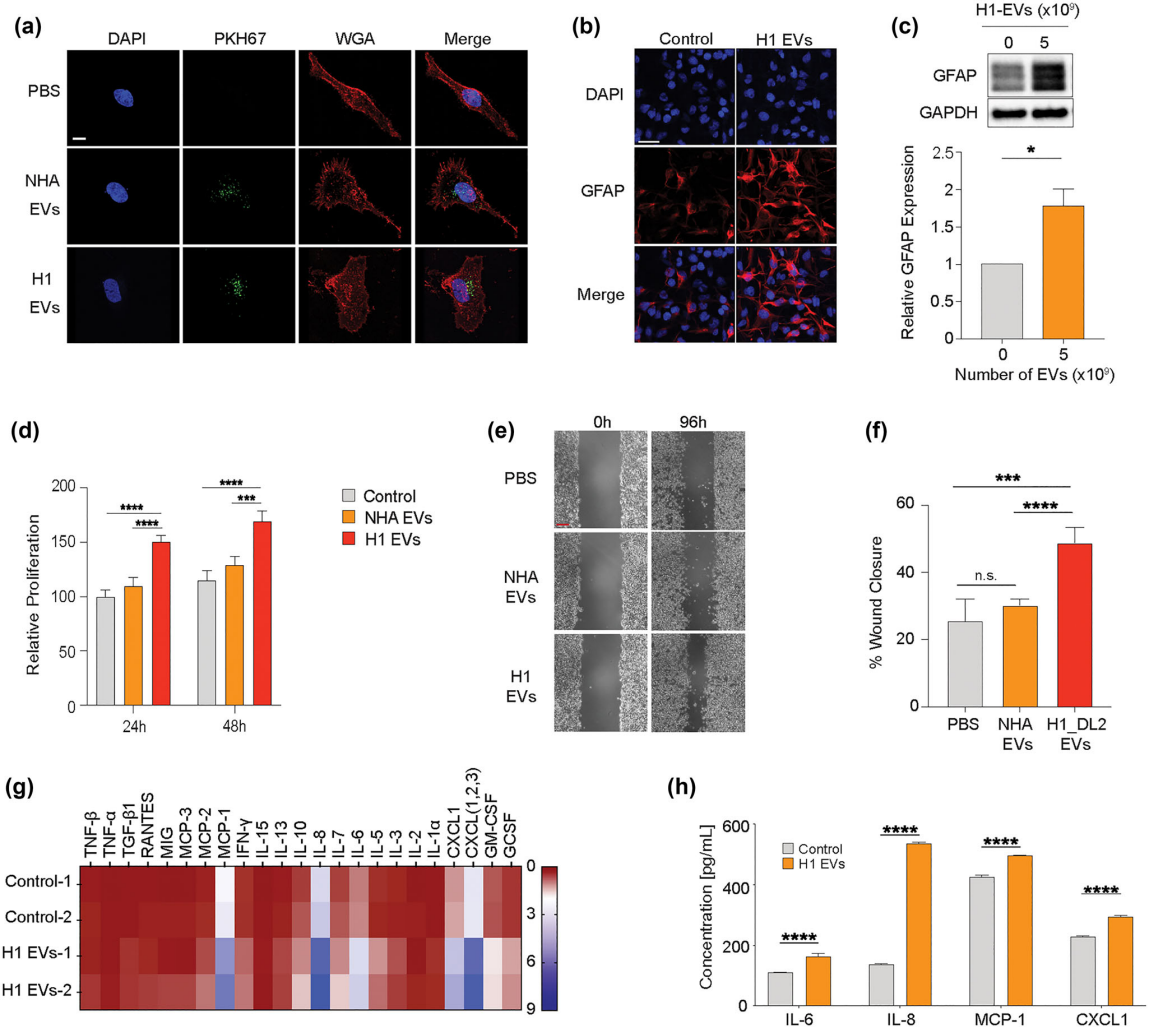
MBM‐derived EVs induce activation in NHA. (a) NHA cultured with 5.0 × 10^9^ EVs of PKH67‐stained (green) NHA‐ or H1‐EVs for 48 h to show uptake. Nuclei stained with DAPI (blue), membrane stained with WGA‐Texas Red (red). Magnification (100X). Scale bar = 10 μm. (b) Representative images of ICC‐stained NHA cells cultured with H1‐EVs or PBS control for 48 h. Merged images of DAPI (blue) and GFAP (red). Magnification 20X. Scale bar = 20 μm. (c) Western blot analysis of GFAP in NHA cells after culturing with 5.0 × 10^9^ H1‐EVs or PBS for 48 h and subsequent quantification. (d) CCK8 proliferation assay of NHA cells cultured with 5.0 × 10^9^ NHA‐ or H1‐EVs over 48 h. (e) Representative micrographs at the start and completion of a 96 h NHA wound healing assay co‐cultured with 5.0 × 10^9^ NHA‐ or H1‐EVs. Scale bar: 300 μm. (f) Quantification of wound healing assay as percentage wound closure after 96 h. (g) Heatmap of a cytokine array of NHA conditioned media after culturing with 5.0 × 10^9^ H1‐EVs or PBS for 48 h. (h) ELISA validation performed on NHA conditioned media after co‐culture with 5.0 × 10^9^ H1‐EVs. ELISA of top four upregulated cytokines from cytokine array, IL‐6, IL‐8, MCP‐1 and CXCL1, were used. n.s.= not significant *p < 0.05, ***p < 0.001, ****p < 0.0001.

Astrocytes have major functions in maintaining BBB integrity and they consequently represent the first cells that the extravasating tumour cells will encounter within the CNS. Reactive astrocytes have been shown to be involved in numerous reciprocal interactions that brain metastatic cells require for their initiation and growth (Zhang et al., 2015). Therefore, we assessed to what extent MBM‐EVs could be taken up by NHAs and if they were activated. For this purpose, we labelled the NHA with Texas red‐conjugated wheat germ agglutinin (WGA‐Texas red) and the MBM‐EVs with the membrane stain PKH67 (green). As shown in Figure 2(a) and Figure S2(a,b), the EVs derived from H1 and H2 MBM cells, as well as from NHAs, were taken up by the NHA cells. To assess a putative NHA activation, the cells were stained for the glial acidic fibrillary acidic protein (GFAP) known to be over‐expressed in reactive astrocytes (Escartin et al., 2021). As shown in Figure 2(b,c), an exposure to H1‐EVs led to an elevated expression of GFAP in the NHAs. This also resulted in an increased NHA cell proliferation (Figure 2d) as well as cell migration (Figure 2e,f). Interestingly, NHA EVs did not cause a stimulation of the NHAs (Figure 2d–f). NHA cells that were activated by H1 EVs where then co‐cultured with H1_DL2 green, fluorescent cells. By counting the fluorescent cells, we observed an increased H1_DL2 cell proliferation (Figure S3a,b).

Previous studies have shown that activated astrocytes display altered cytokine expression levels in the brain (Zhang et al., 2018), which may contribute to MBM progression (Wasilewski et al., 2017). This, however, has not been determined in the context of EVs exposure and the establishment of the brain metastatic niche in melanoma. Therefore, we performed a cytokine array on conditioned media from NHAs that were activated by MBM‐EVs. As shown in Figure 2(g), NHAs exposed to H1‐EVs led to an upregulation of several cytokines that support MBM growth such as IL‐6, IL‐8, MCP‐1 (CCL2) and CXCL1 (Dhawan & Richmond, 2002; Fares et al., 2021). These cytokines were also verified by enzyme‐linked immunosorbent assays (ELISA, Figure 2h, Figure S4). Specifically, we found that neutralizing IL‐8 and IL‐6 activity significantly reversed the increased proliferation in H1 cells (Figure S5). Interestingly, these cytokines have previously been shown to increase melanoma cell migration and invasion through an activation of the mitogen‐activated protein kinase (MAPK) and Notch signalling pathways (Alles et al., 2019; Dhawan & Richmond, 2002).

In conclusion, we show that MBM‐EVs are taken up and activates NHAs, leading to increased proliferation and migration. Moreover, the activated NHAs show an increased expression of cytokines known to support MBM growth.

### 3.3 MiR‐146a‐5p is expressed at high levels in EVs derived from MBMs and has a functional role in NHA activation

MiRNAs are known to be the most abundant RNA cargo in EVs, and it has been confirmed that EV packaged‐miRNAs from tumour cells are involved in a microenvironmental modulation that may support tumour cell proliferation, metastasis, angiogenesis, chemoresistance and immune regulation (Kulkarni et al., 2019; Sun et al., 2018). Detailed knowledge of the EVs cargo provides an avenue for novel clinical biomarker discovery and potential therapeutic intervention. Therefore, we aimed to identify the miRNAs in MBM‐EVs that could be responsible for the observed NHA stimulation.

For this purpose, we performed an Affymetrix array profiling on EVs collected from three MBM cell lines (H1, H2, H3), a cell line derived from a subcutaneous melanoma metastasis (Melmet 1), a lymph node metastasis from melanoma (Melmet 5), and NHA and melanocytes.

**FIGURE 3:**
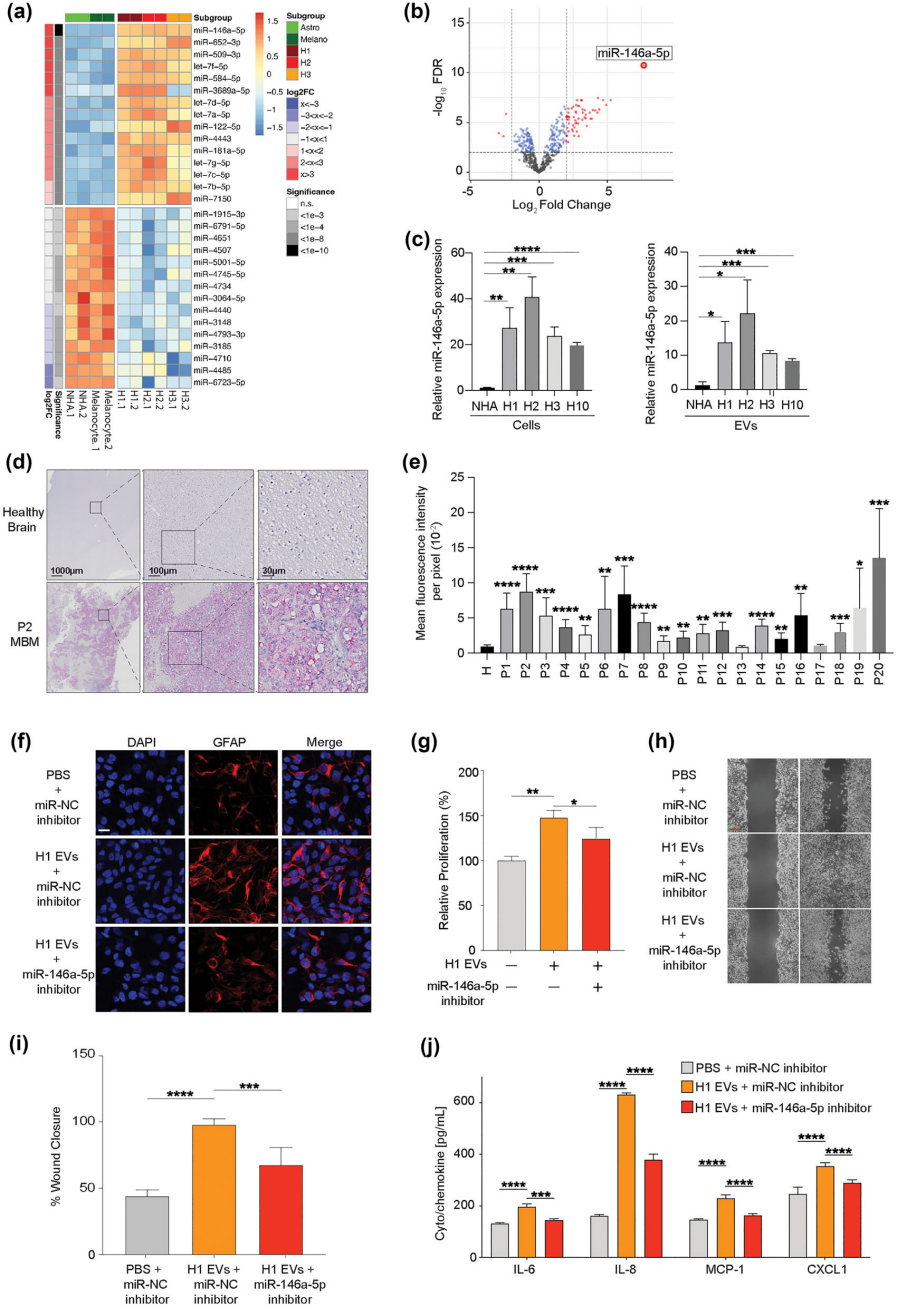
mir‐146a‐5p is significantly upregulated in H1‐EVs compared to normal melanocytes and astrocytes and induces similar responses in NHA cells as H1‐EVs. (a/b) Heatmap of the standardized expression for the top 15 up‐ and down‐regulated miRNAs (a) and a volcano‐plot (b) of differential miRNA expression in EVs derived from healthy (melanocytes and astrocytes) compared to MBM cell lines (H1, H2, H3). miR‐146a‐5p shows stable overexpression in MBM cell lines. (c) qPCR of miR‐146a‐5p expression in EVs and cells across multiple MBM cell lines (H1, H2, H3, H10) and normal cells (NHA). Cells normalized to endogenous control miR‐103, and EVs normalized to spike‐in *C‐elegans* miR‐39‐3p. (d) MiRNAscope in situ hybridization assay of miR‐146a‐5p expression in patient MBM samples compared to healthy brain controls. Red dots indicate successful binding of the miR‐146a‐5p probe, tissues counterstained with haematoxylin (purple). (e) Quantification of miR‐146a‐5p expression across patient samples compared to three pooled healthy brain controls. Data represented as mean fluorescence of 10 representative equal sized areas from each sample ± SE. (f) Representative images of ICC‐stained NHA cells cultured with H1‐EVs treated with miR‐146a‐5p inhibitor or miR‐NC (negative control). Merged images of DAPI (blue) and GFAP (red). Magnification 20X. Scale bar = 20 μm. (g) CCK8 proliferation assay of NHA cells after co‐culture with 5.0 × 10^9^ H1‐EVs in the presence or absence of a miR‐146a‐5p inhibitor. (h) Representative micrographs at the beginning and completion of a 96 h NHA wound healing assay co‐cultured with H1‐EVs with or without miR‐146a‐5p or scramble inhibitor. Scale bar = 300 μm. (i) Quantification of wound healing assay as percentage wound closure after 96 h. J ELISA of IL‐6, IL‐8, MCP‐1 and CXCL1 levels from NHA cell conditioned media after co‐culture with 5.0 × 10^9^ H1‐derived EVs in the presence or absence of miR‐146a‐5p inhibitor. *p < 0.05, **p < 0.01, ***p < 0.001, ****p < 0.0001.

A detailed analysis of differentially expressed miRNAs (MBM‐EVs vs. normal cell‐EVs) revealed that miR‐146a‐5p was by far the most significantly upregulated miRNA across all MBM cell lines (Figure 3a,b).

In patient‐derived cell lines derived from melanoma skin and lymph node metastases (Melmet 1 and Melmet 5), miR‐146a‐5p was elevated compared to the normal samples, but not to the same extent as observed in the MBMs (Figure S6a). RT‐qPCR confirmed a 20‐fold increase of miR‐146a‐5p in three MBM cell lines, and over 10‐fold in their EVs, compared to NHA (Figure 3c). Comparison of miR‐146a‐5p expression between healthy melanocytes and NHA cells showed relatively low expression in both healthy cell types as well as EVs (Figure S6b). Furthermore, an RNA protection assay was performed to assess if miR‐146a‐5p was loaded inside the EVs. Over 65% of miR‐146a‐5p was retained following RNase treatment (Figure S6c).

We initially investigated miR‐146a‐5p by evaluating expression levels in EVs from blood serum from nine healthy volunteers, 26 melanoma patients without MBM and nine melanoma patients with confirmed MBM. No increase of miR‐146a‐5p expression was found in MBM patients versus healthy volunteers, and in fact healthy participants had the highest miR‐146a‐5p in their serum EVs compared to MBM patients (Figure S7a,b).

However, investigating further to confirm a putative role of miR‐146a‐5p in MBMs, in situ hybridization (ISH) was used to assess the expression levels of miR‐146a‐5p in 20 human MBM biopsies. Here, all tumour samples showed elevated expression levels compared to normal brain tissue. Moreover, 18 of the 20 clinical samples revealed a significantly higher expression of miR‐146a‐5p compared to healthy normal brain tissue (Figure 3d,e, Figure S8).

MiR‐146a‐5p has previously been shown to be implicated in melanoma growth and metastasis (Forloni et al., 2014; Pu et al., 2018; Raimo et al., 2016; Zhang et al., 2020); however, its role in EV delivery and niche establishment has not been determined. To determine if miR‐146a‐5p was responsible for the NHA activation mediated by the MBM EVs, NHAs were simultaneously co‐cultured with H1‐EVs and a single stranded RNA‐miR‐146a‐5p inhibitor. We confirmed uptake of the inhibitor into NHAs by confocal microscopy (Figure S9). As shown in Figure 3(f), the inhibitor caused a reduced GFAP expression in NHAs following H1‐EV exposure. Moreover, the inhibitor also reduced the effect of EVs on NHA cell proliferation and migration (Figure 3g–i, Figure S10a). By ELISA, we further show that miR‐146a‐5p inhibition caused reduced expression of IL‐6, IL‐8, MCP‐1 and CXCL1 following exposure of H1‐and H2‐EVs to NHAs (Figure 3j, Figure S10b).

In conclusion, miR‐146a‐5p is expressed in human MBMs and MBM EVs and has a major role in the functional activation of NHAs following MBM EVs exposure.

### 3.4 MiR‐146a‐5p increases Notch signalling through downregulation of NUMB expression

**FIGURE 4:**
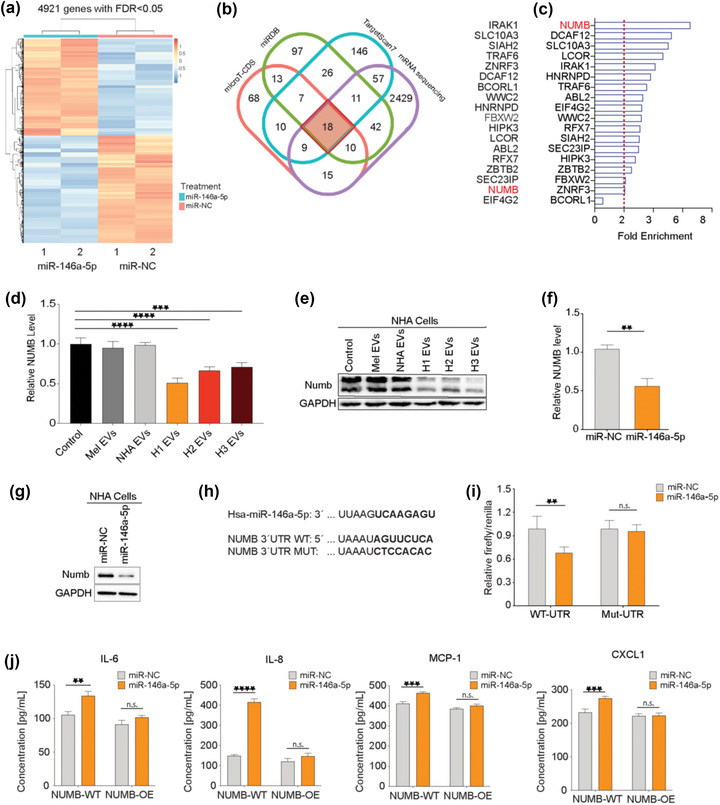
miR‐146a‐5p increased NOTCH signalling through interaction with and downregulation of NUMB expression. (a) Heatmap of significantly differentially expressed mRNA (FDR < 0.05) from NHA cells treated with a miR‐146a‐5p mimic for 48 h and compared to a scrambled control (miR‐NC). (b) Significantly down‐regulated mRNA results were compared to three databases (TargetScan 7.1, miRBD and microT‐CDS) and 18 common genes were found as in situ projected binding partners. (c) mRNA‐pulldown assay followed by qPCR quantification of bound mRNA‐miR‐146a‐5p complexes from the common 18 binding partners. (d) qPCR of NUMB expression in NHA cells after co‐culture with 5.0 × 10^9^ EVs from melanocytes, NHA or MBM cell lines (H1, H2 and H3), normalized with U6 expression. (e) WB of Numb protein expression in NHA cells after co‐culture with 5.0 × 10^9^ EVs from melanocytes, NHA or MBM cell lines (H1, H2 and H3). (f) qPCR of NUMB expression in NHA cells after co‐culture with miR‐146a‐5p mimic or a scrambled control, normalized to U6 expression. (g) Western blot of Numb protein expression in NHA cells after co‐culture with miR‐146a‐5p mimic or a scrambled control. (h) Sequence of miR‐146a‐5p 3p binding site, and the comparison of NUMB 3′UTR‐wild type (WT) and mutated (MUT) region. (i) Dual luciferase assay on the WT or MUT 3′UTR region with miR‐146a‐5p or a scrambled control in NHA cells, represented as the relative firefly to *Renilla* luciferase activity. (j) ELISA of top four upregulated cytokines released by NHA cells with WT or overexpression of NUMB in the presence of miR‐146a‐5p mimic or control. n.s.= not significant, **p < 0.01, ***p < 0.001, ****p < 0.0001.

To assess how miR‐146a‐5p affects NHA activation at the transcription level, mRNA sequencing was performed on NHA cells exposed either to miR‐146a‐5p mimics or a scrambled control. MiR‐146a‐5p overexpression via a miRNA mimic led to 2330 differentially upregulated and 2591 differentially downregulated mRNAs (Figure 4a). We then performed online queries using databases predicting potential biological targets of miR‐146a‐5p (TargetScan, miRDB and microT‐CDS online tools; Figure S11). By combining the downregulated sequencing data with the online queries, we identified 18 overlapping potential binding partners of miR‐146a‐5p (Figure 4b). A pulldown assay of NHA lysates followed by RT‐qPCR was performed to quantify the enrichment of mRNA‐miR‐146a‐5p complexes. As shown in Figure 4(c), NUMB was the most abundant binding partner, having around 6‐fold more bound complexes compared to the scrambled control.

The Numb protein is an inhibitor in the Notch signalling pathway where it acts as a tumour suppressor. It has previously been shown that miR‐146a‐5p down‐regulates NUMB leading to melanoma initiation and progression by activating the Notch signalling pathway (Forloni et al., 2014). Furthermore, data from The Cancer Genome Atlas (TCGA) also show a correlation between poor survival in melanoma patients and low NUMB expression levels (Hristova et al., 2021).

Based on this information, we aimed to study the putative role of EV enriched miR‐146a‐5p in NUMB inhibition in the context of MBM. As shown in Figure 4(d), RT‐qPCR analysis revealed a significant down‐regulation of Numb mRNA expression in NHA cells following a co‐culture with H1‐, H2‐ and H3‐EVs. In contrast, EVs from melanocytes and NHAs did not have any effect. These findings were further substantiated at the protein level by WB analysis (Figure 4e). Moreover, the same results were observed by RT‐qPCR and WB and in NHA cells after co‐culture with a miR‐146a‐5p mimic (Figure 4f,g) indicating a causal relationship between miR‐146a‐5p and NUMB.

To further verify a putative target relationship between miR‐146a‐5p and NUMB, NHA cells were transfected with luciferase constructs containing either wild‐type (WT) NUMB or a mutated version (Mut) of the miR‐146a‐5p binding site (Figure 4h). As shown in Figure 4(i), following an addition of miR‐146a‐5p mimic, luciferase activity was significantly reduced. In comparison, no significant difference was found between miR‐146a‐5p and miR‐NC in mutated 3′ region samples, indicating a specific miR‐146a‐5p to NUMB binding.

In NHA, we performed a NUMB overexpression (Figure S12a) to determine the effects of miR‐146a‐5p on the previously studied cytokine production. An increased production of IL‐6, IL‐8, MCP‐1 and CXCL1 were observed in the wild type NHA (NUMB‐WT) when exposed to the miR‐146a‐5p mimic, whereas NHA overexpressing NUMB (NUMB‐OE) did not show the same effect (Figure 4j) suggesting NUMB involvement in NHA activation/dysregulation.

In summary, NUMB is a direct miR‐146a‐5p target in NHAs, and its overexpression abrogates their cytokine production.

### 3.5 MiR‐146a‐5p increases Notch expression levels and downstream proteins in NHAs

**FIGURE 5:**
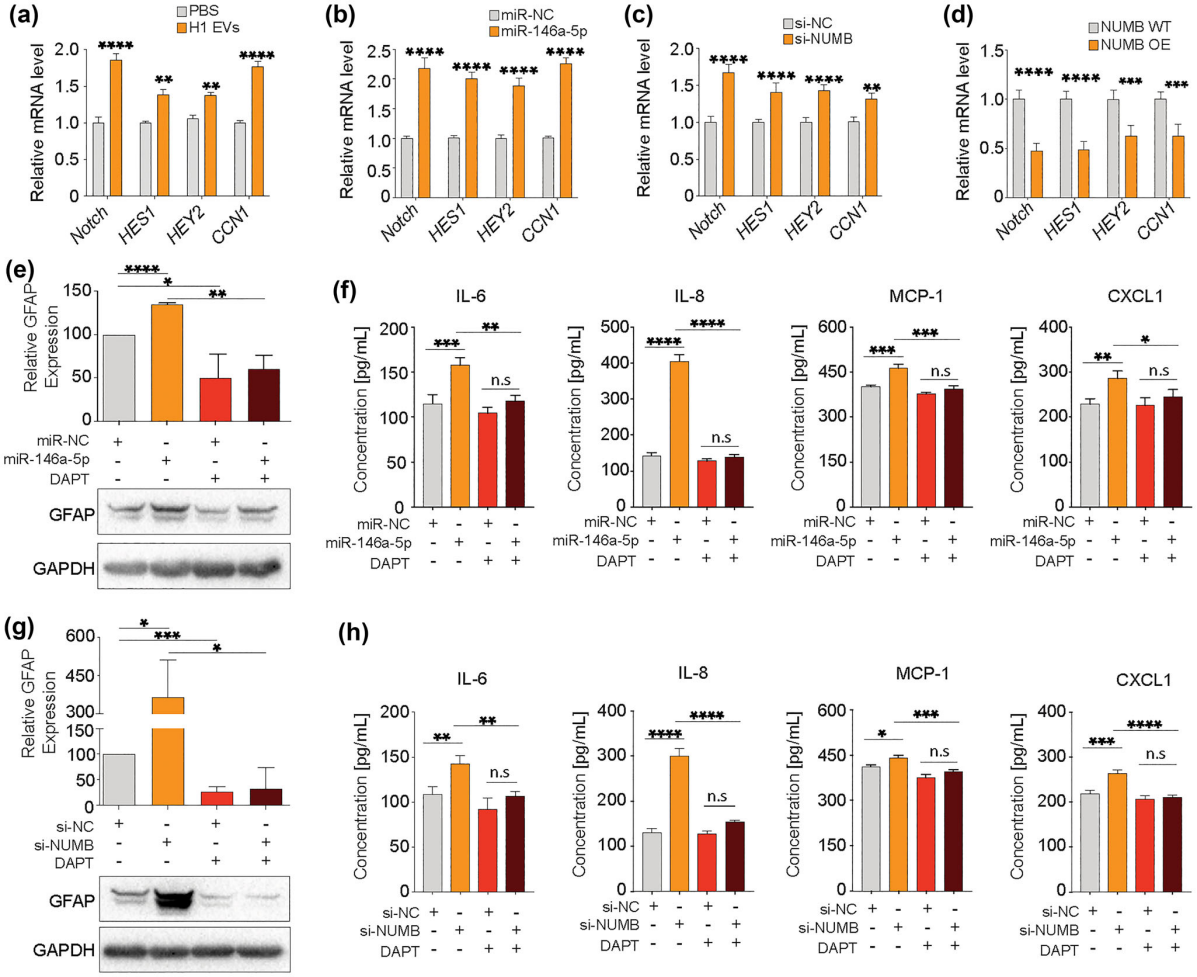
miR‐146a‐5p influences the activation of astrocytes through the upregulation of the Notch pathway via NUMB inhibition. (a–d) qPCR of relative mRNA expression of proteins involved in the Notch pathway from NHA cells after treatment with H1‐derived EVs or PBS control, miR‐146a‐5p mimic or a scrambled control, NUMB siRNA or a scrambled control, and NUMB OE or WT control. (e) Western blot analysis of GFAP expression in NHA cells after treatment with miR‐146a‐5p mimic and/or Notch inhibitor DAPT and associated quantification. (f) ELISA of top four upregulated cytokines released by NHA cells after treatment with miR‐146a‐5p mimic with and without the Notch pathway inhibitor DAPT. (g) Western blot analysis of GFAP expression in NHA cells after treatment with NUMB siRNA and/or DAPT associated quantification. (h) ELISA of top four up regulated cytokines released by NHA cells after treatment with NUMB siRNA and/or DAPT. All data displayed from three independent experiments. n.s.= not significant, *p < 0.05, **p < 0.01, ***p < 0.001, ****p < 0.0001.

Next, we studied the expression of downstream proteins in the Notch signalling pathway. Expression levels of Notch, HES1, HEY2 and CCN1 increased in NHAs following addition of either H1‐EVs or the miR‐146‐5p mimic (Figure 5a,b). NUMB inhibition via siRNA (Figure S12b) verified a causal relationship between NUMB and proteins of the Notch signalling pathway, as seen with increased expression of Notch, HES1, HEY2, and CCN1, compared to vector control (Figure 5c). In NHA cells overexpressing NUMB, the expression levels decreased, as assessed by qPCR (Figure 5d).

The γ‐secretase inhibitor DAPT (N‐[N‐(3, 5‐difluorophenacetyl)‐l‐alanyl]‐s‐phenylglycinet‐butyl ester) is known to indirectly block Notch signalling activity (Geling et al., 2002). To further assess the effect of Notch signalling following miR‐146a‐5p mimic‐induced NHA activation (assessed by GFAP expression), the cells were exposed to DAPT. As shown in Figure 5(e), the miR‐146a‐5p mimic increased GFAP expression levels significantly, as compared to NHA exposed to a scrambled miR negative control (miR‐NC). DAPT treatment lowered GFAP levels regardless of whether miR‐146a‐5p mimic or a scrambled miR‐NC control was added to the cultures.

Thereafter, we determined the expression levels of cytokines (IL‐6, IL‐8, MCP‐1 and CXCL1) after miR‐146a‐5p and/or DAPT exposure to NHA cells. As expected, all four cytokines were elevated following miR‐146a‐5p mimic exposure, while after treatment with either DAPT alone or in combination with miR‐146a‐5p mimic cytokine expression remained the same as the control (Figure 5f). NHA were activated after silencing NUMB, as seen by elevated GFAP expression. However, this effect was reversed after treatment with DAPT alone or combined with si‐NUMB (Figure 5g). The same effect was seen in a similar experiment where silencing of NUMB was induced in NHA cells with or without DAPT treatment. Upon silencing of NUMB, increased production of all four cytokines were observed, while combined treatment with DAPT decreased cytokine expression to normal levels (Figure 5h).

In summary, miR‐146a‐5p alters cytokine expression via NUMB silencing and dysregulates downstream proteins in the Notch signalling pathway including HES1, HEY2 and CCN1 suggesting a dysregulation of the overall Notch pathway.

### 3.6 Mir‐146a‐5p knockdown (KD) in MBM cells decreases tumour burden and increases animal survival

**FIGURE 6:**
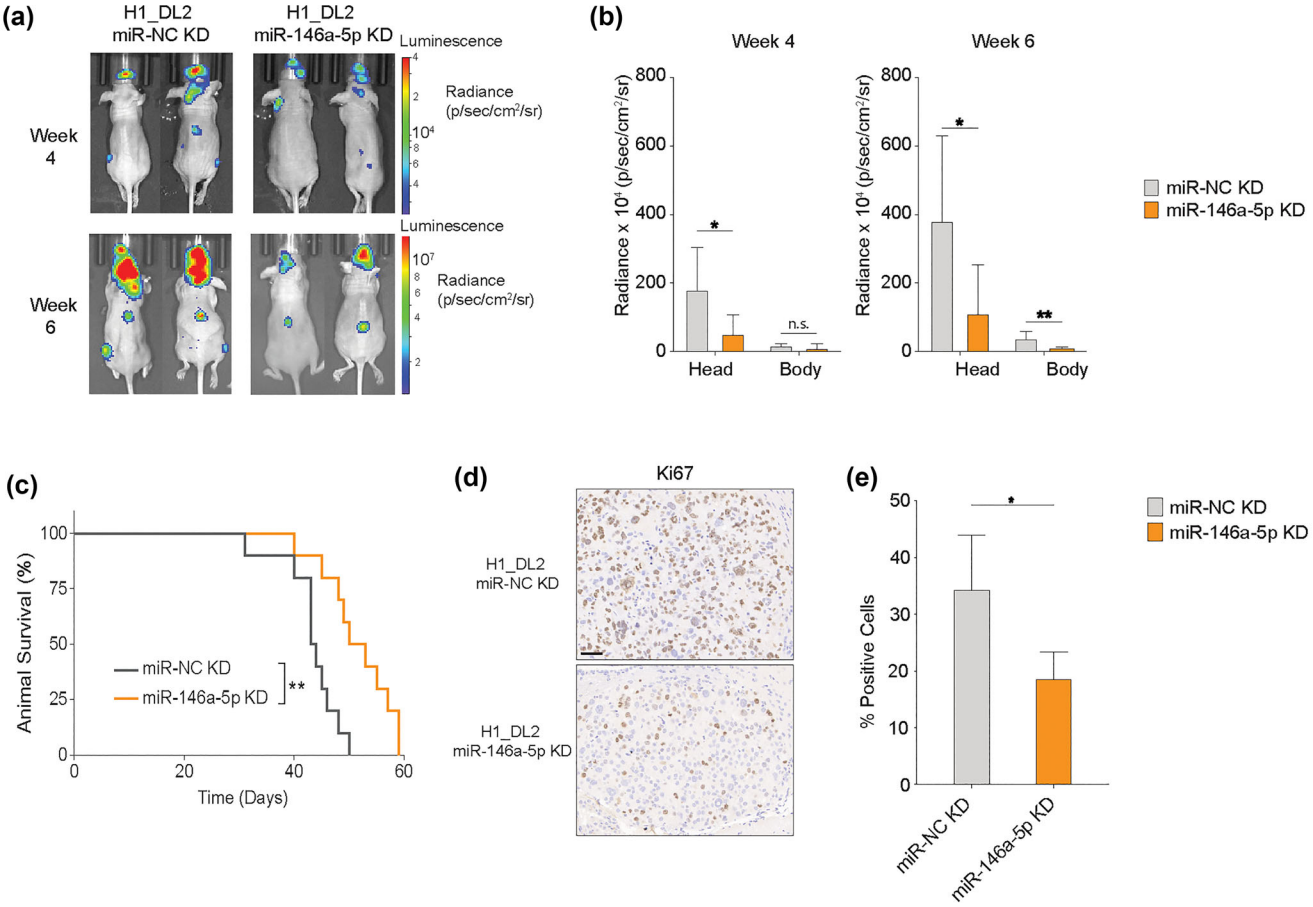
miR‐146a‐5p knockdown in MBM cells significantly decreased tumour burden and increased survival in a mouse brain metastasis model. (a) 5 × 10^5^ H1_DL2 miR‐NC KD (control) or H1_DL2 miR‐146a‐5pKD cells were injected intracardially into mice. Tumour burden was evaluated at week 4 and 6 with IVIS bioluminescent imaging. *n* = 10 mice per group. (b) Tumour distribution in head and body were quantified at week 4 and 6 by the average radiance in photons/s/cm^2^/steradia (p/s/cm^2^/sr). (c) Kaplan–Meier survival curves calculated for all animals in the treatment study. (d) Representative images of Ki67 staining of FFPE sections of brain tumour tissue from animal test subjects after sacrificing. Scale bar = 50 μm. (e) Quantification of Ki67 staining as a percentage of total cells in each tumour. n.s.= not significant, *p < 0.05, **p < 0.01.

A proof‐of‐concept study was performed with a miR‐146a‐5p knockdown model to investigate its effect on MBM development in vivo. MiR‐146a‐5p was knocked down by lentiviral transduction of a sponge mRNA in H1_DL2 cells (miR‐146a‐5p KD, Figure S13) and injected i.c. into female nude mice. As a control, mice were injected i.c. with H1_DL2 cells transduced with a scrambled miR‐NC vector. Bioluminescence imaging showed a significantly reduced MBM burden at weeks 4 and 6 in mice injected with miR‐146a‐5p KD cells, as compared to control animals (Figure 6a,b). Furthermore, at week 6, a reduced tumour burden in the body of mice injected with miR‐146a‐5p KD cells was observed (Figure 6b). miR‐146a‐5p KD mice survived significantly longer than control mice (Figure 6c), and the miR‐146a‐5p KD tumours displayed a lower Ki67 expression indicating less tumour proliferation (Figure 6d,e).

Taken together, miR‐146a‐5p KD reduced MBM tumour burden and increased survival in a mouse model of human MBM, indicating its involvement in MBM development.

### 3.7 Deserpidine inhibits miR‐146a‐5p activity and suppresses MBM development

**FIGURE 7:**
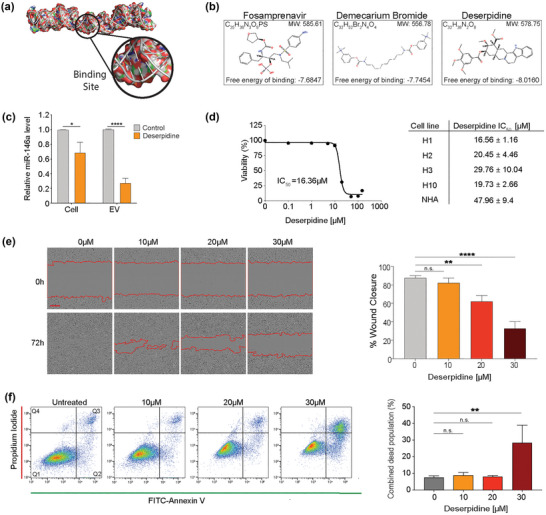
Deserpidine reduces the expression of miR‐146a‐5p in vitro and reduces the viability and proliferation of MBM cells. (a) Schematic representation of the 3D structure of the miR‐146a‐5p binding site for high‐throughput molecular docking analysis. (b) Schematic representation of the top 3 binding partners of miR‐146a‐5p available for purchase and predicted to cross the blood brain barrier. (c) qPCR analysis of miR‐146a‐5p expression in H1 cells and EVs after deserpidine treatment. (d) Representative IC_50_ survival curve of H1 cells after treatment with increasing drug concentrations (0.01–150 μM) for 72 h and corresponding deserpidine IC_50_ doses of all MBM cell lines and NHA. (e) Representative images from wound healing assay of H1 cells after treatment with various concentrations of deserpidine and quantification of % wound closure at 72 h. Scale bar = 300 μm. (f) Annexin V‐FITC and PI staining to assess apoptosis by flow cytometry in H1 cells treated with increasing concentrations of deserpidine for 72 h and corresponding quantification of live cell and apoptotic cell populations (Q2 + Q3) represented as percentage of total cell population. All data displayed from three independent experiments. Quadrant 1: Live cells. Quadrant 2: Early apoptosis. Quadrant 3: Late apoptosis. Quadrant 4: Necrosis. n.s.= not significant, *p < 0.05, **p < 0.01, ****p < 0.0001. *N* = 3.

In order to identify potential drugs that could suppress miR‐146a‐5p activity, we performed an *in silico* structure modelling to predict the 3‐dimensional structure of the hairpin loop of the pre‐miR‐146a (Figure 7a), followed by a virtual screening of compounds with the potential to bind to the miR‐146a‐5p 3D‐modelled structure (Figure 7b). The top three candidates predicted to cross the BBB were deserpidine, demecarium bromide (demecarium) and fosamprenavir. These drugs were chosen for further functional in vitro experiments. Both deserpidine and demecarium reduced miR‐146a‐5p levels significantly in both H1 cells and H1‐EVs, while fosamprenavir did not alter the miRNA levels significantly (Figure 7c, Figure S14a). A viability screening on H1, H2, H3 and H10 cells determined the IC_50_ doses to be much lower for deserpidine (16.56–29.76 μM) (Figure 7d) than demecarium (97.79–271.33 μM) (Figure S14b,c), thus deserpidine was chosen for subsequent experiments.

Deserpidine is an ester alkaloid derived from Rauwolfa canescens and has been used in the treatment of psychosis and hypertension (Zhang et al., 2009). A dose‐dependent inhibition of cell migration was observed in deserpidine‐treated H1 cells (Figure 7e), as well as a dose‐dependent induction of apoptosis in H1 cells (Figure 7f). Additionally, EVs derived from deserpidine‐treated H1 cells did not activate NHAs (Figure S15), further confirming the role of MBM‐EVs in NHA activation.

Here, deserpidine decreased miR‐146a‐5p expression levels in H1 MBM cells and in H1‐EVs, subsequently leading to induction of apoptosis and inhibition of cell viability and migration in these cells.

### 3.8 Deserpidine inhibits MBM tumour burden and increases animal survival

**FIGURE 8:**
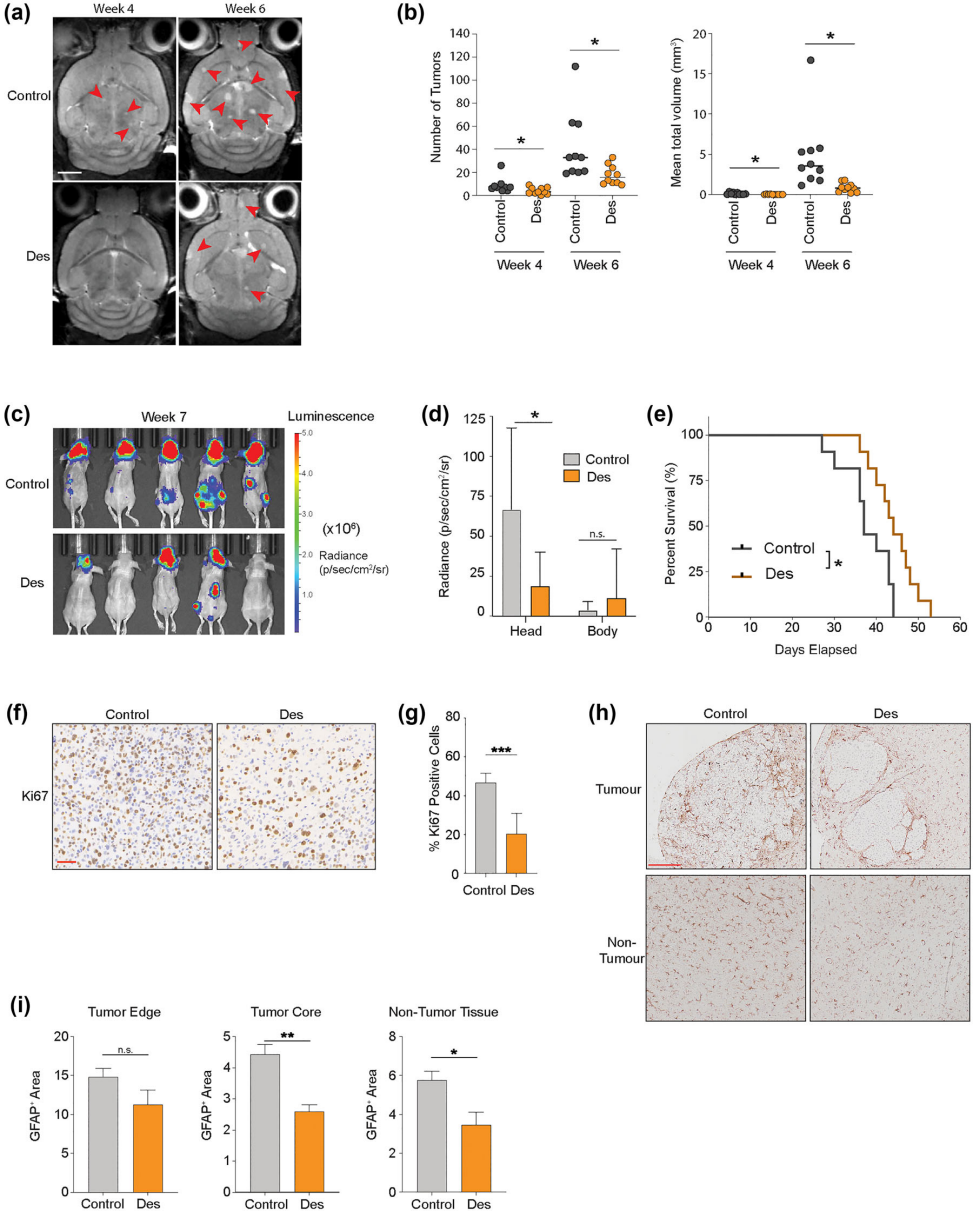
Deserpidine reduces tumour burden and increases mice overall survival in vivo. (a) Development of brain metastasis assessed by T2 weighted MRI at week 4 and 6 after intracardial injection of MBM H1_DL2 cells. Mice were either treated with 0.15 mg/kg deserpidine or solvent 3 days per week. *n* = 10 mice per group. (b) Quantification of the numbers and volumes of brain metastasis in 0.15 mg/kg deserpidine‐treated and control animals. (c) Development of tumour burden evaluated week 4 and 6 with IVIS bioluminescent imaging after i.c. injection of MBM H1_DL2 cells. Mice were either treated with 0.5 mg/kg deserpidine or solvent every 3 days. *n* = 10 mice per group. (d) tumour distribution in head and body were quantified by the average radiance in photons/s/cm^2^/steradia (p/s/cm^2^/sr). (e) Kaplan–Meier survival curves calculated for all animals in the second treatment study. (f) Representative images of Ki67 staining of formalin fixed paraffin embedded (FFPE) sections of brain tumour tissue from animal test subjects after sacrificing. Scale bar: 50 μm. (g) Quantification of Ki67 staining as a percentage of total cells in each tumour. (h) Representative images of GFAP staining of FFPE sections of brain tumour tissue and brain non‐tumour tissues from animal test subjects after sacrificing. Scale bar = 200 μm. (i) Quantification of GFAP staining as a percentage of intensity of GFAP in tumour edge, tumour core and non‐tumour tissue areas. Data are displayed as mean ± SEM. Groups were compared using the Welch *t*‐test. *p < 0.05, **p < 0.01, ***p < 0.001.

To determine the preclinical potential of deserpidine, female NOD/SCID mice were injected i.c. with 5.0 × 10^5^ H1_DL2 cells and treated with either deserpidine or solvent only as control for 6–7 weeks. MRI showed that in mice treated with 0.15 mg/kg deserpidine, the numbers and volumes of MBM tumours were significantly reduced, compared to control mice (no treatment) (Figure 8a,b).

In a second experiment, bioluminescence imaging confirmed that mice injected with 0.5 mg/kg deserpidine had less tumour burden, compared to the control animals. No effects were observed on extracranial metastasis (Figure 8c,d). A Kaplan–Meyer survival plot showed increased animal survival in deserpidine treated mice, compared to controls (Figure 8e). Cell proliferation was reduced in MBM tumours from deserpidine treated mice compared to control mice, as indicated by Ki67 expression (Figure 8f,g). GFAP staining showed a significant reduction of GFAP+ cells within the tumour mass and in normal brain tissue after deserpidine treatment, as well as a trend towards reduction in normal tissue close to the tumour edge (Figure 8h,i). Taken together, these results suggest that deserpidine shows inhibition of tumour growth which further adds to inhibition of miR146a‐5p and therefore should be further exploited in a clinical context of MBM.

## 4 DISCUSSION

Despite multimodal treatment, brain metastasis is a common complication in melanoma patients with advanced disease (Achrol et al., 2019). Thus, an improved molecular understanding of the metastatic process with the aim of discovering new adjuvant treatments is necessary. It has previously been shown that brain metastasis development is mediated by complex interactions between tumour cells and normal cells, facilitated in part by the transfer of EVs to establish a metastasis‐promoting environment (Gowda et al., 2020; Tamas et al., 2022). To our knowledge this is the first study showing that MBM‐EVs pretreatment increases brain metastasis burden in mice (Figure 1e,f), possibly due to increased transmigration of tumour cells through the BBB or improved survival of those cells already transmigrated. The importance of astrocytes in development of both primary and secondary neural malignancies is well documented, but the molecular mechanisms of astrocyte‐tumour interactions are only just being discovered (Burn et al., 2021; Zou et al., 2019).

In the present study, we demonstrate that healthy astrocytes are remodelled via transfer of EV‐packaged miR‐146a‐5p from MBM cells, which increases Notch signalling, stimulating MBM development. We also show, in a biological and therapeutic context, that a reduction of miR‐146a‐5p activity reduces MBM formation.

For melanomas, reports regarding the role of miR‐146a‐5p in metastatic development is conflicting. In a xenograft model by Hwang and colleagues, a loss of miR‐146a‐5p expression was seen in melanoma cells, where its overexpression reduced migration and invasion. In contrast, Pu and colleagues found that miR‐146a‐5p increased cell migration and invasion in melanomas (Hwang et al., 2012; Pu et al., 2018). Here, we show that miR‐146a‐5p is highly expressed across a number of human MBM cell lines, as well as in their secreted EVs, compared to healthy astrocytes and melanocytes. In addition, significantly higher miR‐146a‐5p levels were seen in clinical MBM samples, compared to healthy brain tissue (Figure 3i,j). In this context, prior clinical studies suggest that miR‐146a‐5p is overexpressed in both primary and metastatic lesions, but according to an in vivo melanoma progression model, it is reduced in circulating tumour cells (CTCs), indicating a site‐specific mechanism of action (Dika et al., 2020; Raimo et al., 2016). This is in line with our findings, where we found low levels of miR‐146a‐5p in all melanoma patient blood EV samples compared to healthy individuals, but increased miR‐146a‐5p levels in metastasis samples, with the highest expression levels seen in MBM cell lines and clinical MBM samples. Our initial screening of human serum samples indicates that miR‐146a‐5p is not a suitable candidate as a liquid biopsy biomarker; however, this would need to be evaluated in a larger panel of blood samples. Given this, our data suggests that miR‐146a‐5p has a role in MBM‐EVs by activating astrocytes that stimulates CTCs once they have reached the secondary organ.

A crosstalk between activated astrocytes and tumour cells in the brain may stimulate the progression of both primary and secondary brain tumours (Heiland et al., 2019; Zhang et al., 2015; Zou et al., 2019) through paracrine signalling as a main source of communication where cancer‐induced cytokine profiles in astrocytes stimulate tumour growth and also protect them against immune responses (Heiland et al., 2019). Here we show that an increased production of key cytokines (IL‐6, IL‐8, MCP‐1 and CXCL1), known to facilitate transmigration of breast cancer cells across the BBB (Fares et al., 2021), were increased following NHA exposure to MBM‐EVs or miR‐146a‐5p mimic. In melanoma, these secreted factors have all been found to support growth and dissemination, as corroborated by our findings in Fig S5. IL‐6 induced production of glutathione has been shown to stimulate MBM (Obrador et al., 2011) by triggering MMP‐2 enzymatic activity in the tumour microenvironment (Rossi et al., 2018). Augmented IL‐8 levels have been shown to increase cell migration, invasion, and adhesion capacities of metastatic melanoma cells by activating the MAPK signalling pathway (Liu et al., 2012). Compared to primary melanomas, MCP‐1, and its corresponding receptors CCR2 and CCR4, have been shown to be overexpressed in MBM. An evoked MCP‐1 expression and an altered astrocyte secretome has been reported to enhance MBM proliferation, migration, and invasion (Pozzi et al., 2022). Moreover, increased astrocyte secretion of MCP‐1 can also contribute to an increased BBB permeability that may facilitate MBM establishment (Stamatovic et al., 2003; Weiss et al., 1998; Yao & Tsirka, 2014). Further, CXCL1 has been shown to constitutively increase expression of NF‐κB in melanoma which contribute to melanoma progression via angiogenesis (Dhawan & Richmond, 2002). All these cytokines were triggered in NHAs following MBM‐EV exposure, which suggest that they have a prominent role in MBM formation. The exact mechanisms on how they stimulate MBM progression remains to be elucidated.

Mechanistically we show, using *in silico* and in vitro methods, that NUMB, an inhibitory protein in the Notch signalling pathway, is a binding partner of miR‐146a‐5p in NHAs. MiR‐146a‐5p binding resulted in a targeted silencing of the Numb protein, with a subsequent activation of the several downstream signalling proteins and transcription factors associated with the Notch signalling pathway. Increased Notch signalling can have several effects on cancer development. This includes, among others, apoptosis inhibition, angiogenesis, induction of epithelial to mesenchymal transition (EMT) and formation of brain metastasis (Xing et al., 2013).

Numb has previously been described as a miR‐146a‐5p target in melanoma (Forloni et al., 2014; Raimo et al., 2016). Our research importantly shows an upregulation of the Notch pathway in surrounding astrocytes in vitro via EV transfer of miR‐146a‐5p from melanoma cells, that subsequently stimulates MBM development. To substantiate these results, we performed an in vivo proof‐of‐concept study, by injecting miR‐146a‐5p KD MBM cells i.c. using a reproducible and predictive human MBM animal model (Sundstrøm et al., 2013). A significant decrease in the subsequent brain metastatic burden with increased animal survival was observed after KD, supporting the important role of miR‐146a‐5p in MBM development.

Drugs targeting brain metastasis specifically are not available, and systemic therapies are usually ineffective (Kim et al., 2018). To find such drugs is a challenging task due to, among others, problems associated with BBB penetrance. From a therapeutic viewpoint, repurposing of drugs is an attractive strategy, as the time needed to bring such drugs to the clinic is shorter, less investments are needed, and the risk of failure is lower (Pushpakom et al., 2019). Using targeted *in silico* analysis, we screened commercially available compounds that could potentially bind to the 3D‐modelled structure of miR‐146a‐5p, thus inhibiting its transcription. Subsequent in vitro and in vivo studies showed that deserpidine reduced the brain metastatic tumour burden, resulting in an inhibited MBM development and increased animal survival. Brain tumours dissected from these mice were found to have less penetrance of GFAP+ cells into the tumour mass, and in the overall brain, possibly indicating a reduced migration/invasion ability of the astrocytes via miR‐146a‐5p inhibition.

In conclusion, we show that MBM‐EV‐transferred miR‐146a‐5p is an important driver of MBM niche establishment, via the induction of a cancer‐promoting activated astrocyte phenotype where Notch signalling, and cytokine production are elevated, leading to a feedback loop encouraging MBM growth. Through this novel discovery, we were able to inactivate the miRNA through both KD or deserpidine treatment and thereby decrease MBM growth in vivo. These findings encourage additional assessment of the role of MBM‐EVs‐stromal interactions in metastasis, and a further evaluation of deserpidine as an adjuvant treatment for patients with MBM or for melanoma patients at risk of developing brain metastasis.

## AUTHOR CONTRIBUTIONS

**Emma Rigg**: Data curation; formal analysis; investigation; methodology; writing—original draft; writing—review and editing. **Jiwei Wang**: Data curation; formal analysis; investigation; methodology; writing—original draft; writing—review and editing. **Zhiwei Xue**: Data curation; formal analysis; investigation; writing—review and editing. **Taral R. Lunavat**: Conceptualization; data curation; formal analysis; methodology; writing—review and editing. **Guowei Liu**: Data curation; formal analysis; investigation; writing—review and editing. **Tuyen Hoang**: Data curation; formal analysis; investigation; writing—review and editing. **Himalaya Parajuli**: Data curation; formal analysis; investigation; writing—review and editing. **Mingzhi Han**: Data curation; formal analysis; investigation; writing—review and editing. **Rolf Bjerkvig**: Conceptualization; data curation; formal analysis; funding acquisition; investigation; supervision; writing—original draft; writing—review and editing. **Petr V. Nazarov**: Data curation; formal analysis; writing—review and editing. **Nathalie Nicot**: Data curation; formal analysis; writing—review and editing. **Stephanie Kreis**: Data curation; formal analysis; writing—review and editing. **Christiane Margue**: Data curation; formal analysis; writing—review and editing. **Miléne Tetsi Nomigni**: Data curation; formal analysis; writing—review and editing. **Jochen Utikal**: Data curation; formal analysis; writing—review and editing. **Hrvoje Miletic**: Data curation; formal analysis; writing—review and editing. **Terje Sundstrøm**: Data curation; formal analysis; writing—review and editing. **Lars A. R. Ystaas**: Formal analysis; writing—review and editing. **Xingang Li**: Conceptualization; funding acquisition; supervision; writing—review and editing. **Frits Thorsen**: Conceptualization; data curation; formal analysis; funding acquisition; investigation; project administration; supervision; writing—original draft; writing—review and editing. All authors performed the quality assurance of the paper and reviewed and approved the final versions of the manuscript and the figures.

## CONFLICT OF INTEREST STATEMENT

The authors declare that there are no conflicts of interest.

## Supporting information

Supplementary Information

Supplementary Information

Supplementary Information

Supplementary Information

Supplementary Information

Supplementary Information

Supplementary Information

Supplementary Information

Supplementary Information

Supplementary Information

Supplementary Information

Supplementary Information

Supplementary Information

Supplementary Information

Supplementary Information

Supplementary Information

Supplementary Information

Supplementary Information

Supplementary Information

Supplementary Information
