# Supplementary figures and images for: Inhibition of extracellular vesicle‐derived miR‐146a‐5p decreases progression of melanoma brain metastasis via Notch pathway dysregulation in astrocytes
Source: J Extracell Vesicles. 2023 Sep 27;12(10):12363. doi: 10.1002/jev2.12363 (PMC10533779; doi:10.1002/jev2.12363)

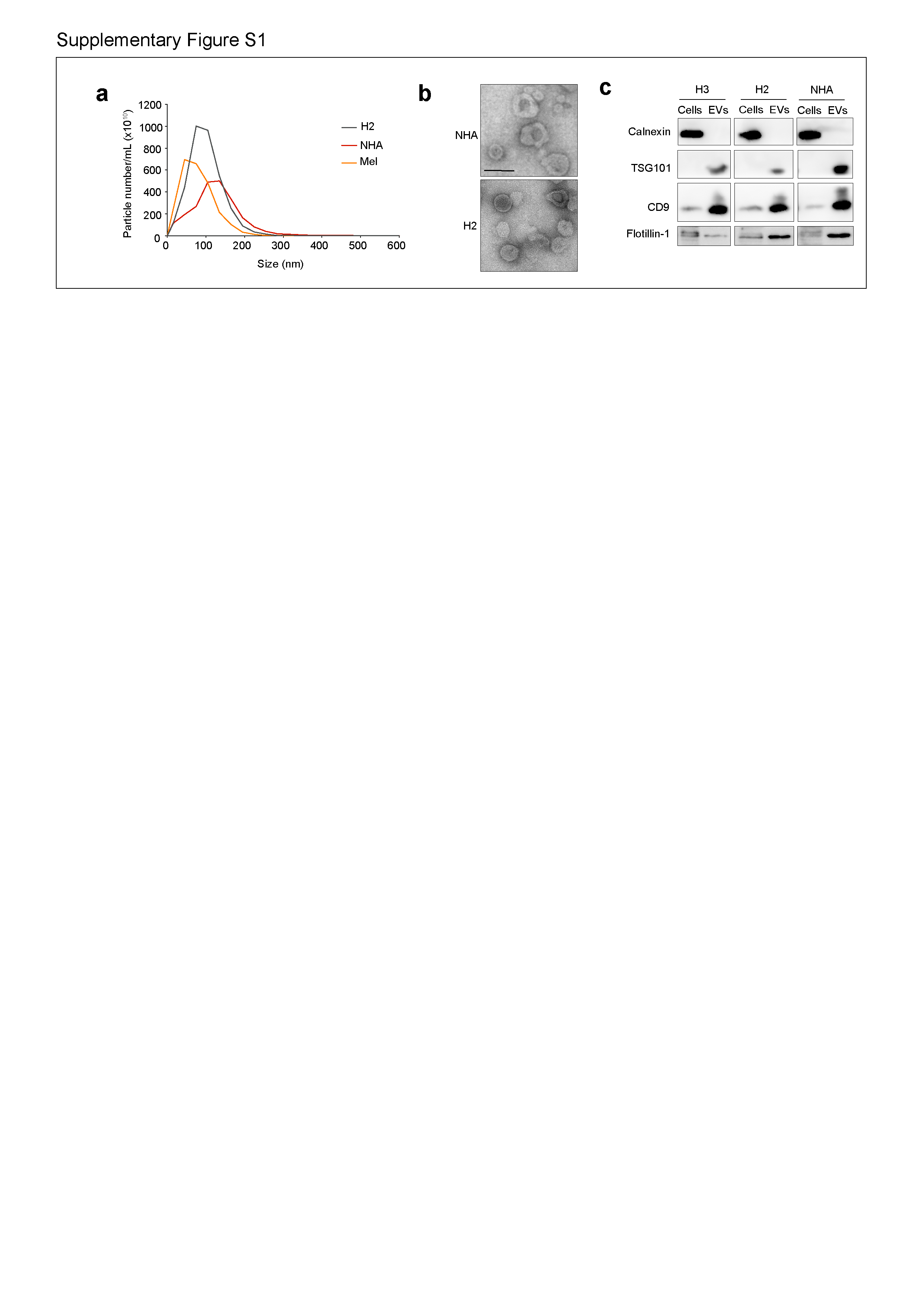

Supplement: Supplementary file 1 — Supplementary Information [file JEV2-12-12363-s017.tif]

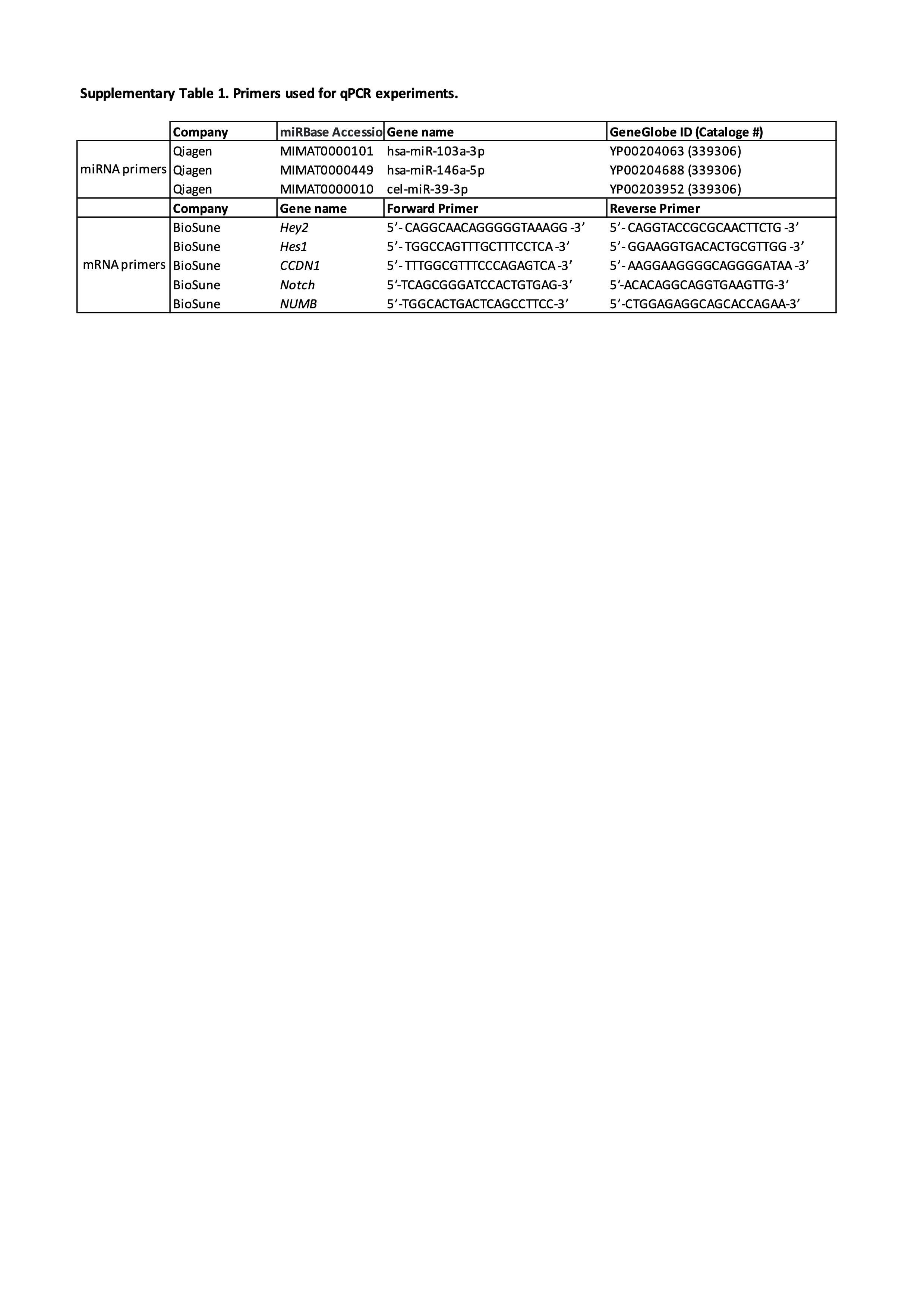

Supplement: Supplementary file 2 — Supplementary Information [file JEV2-12-12363-s012.tiff]

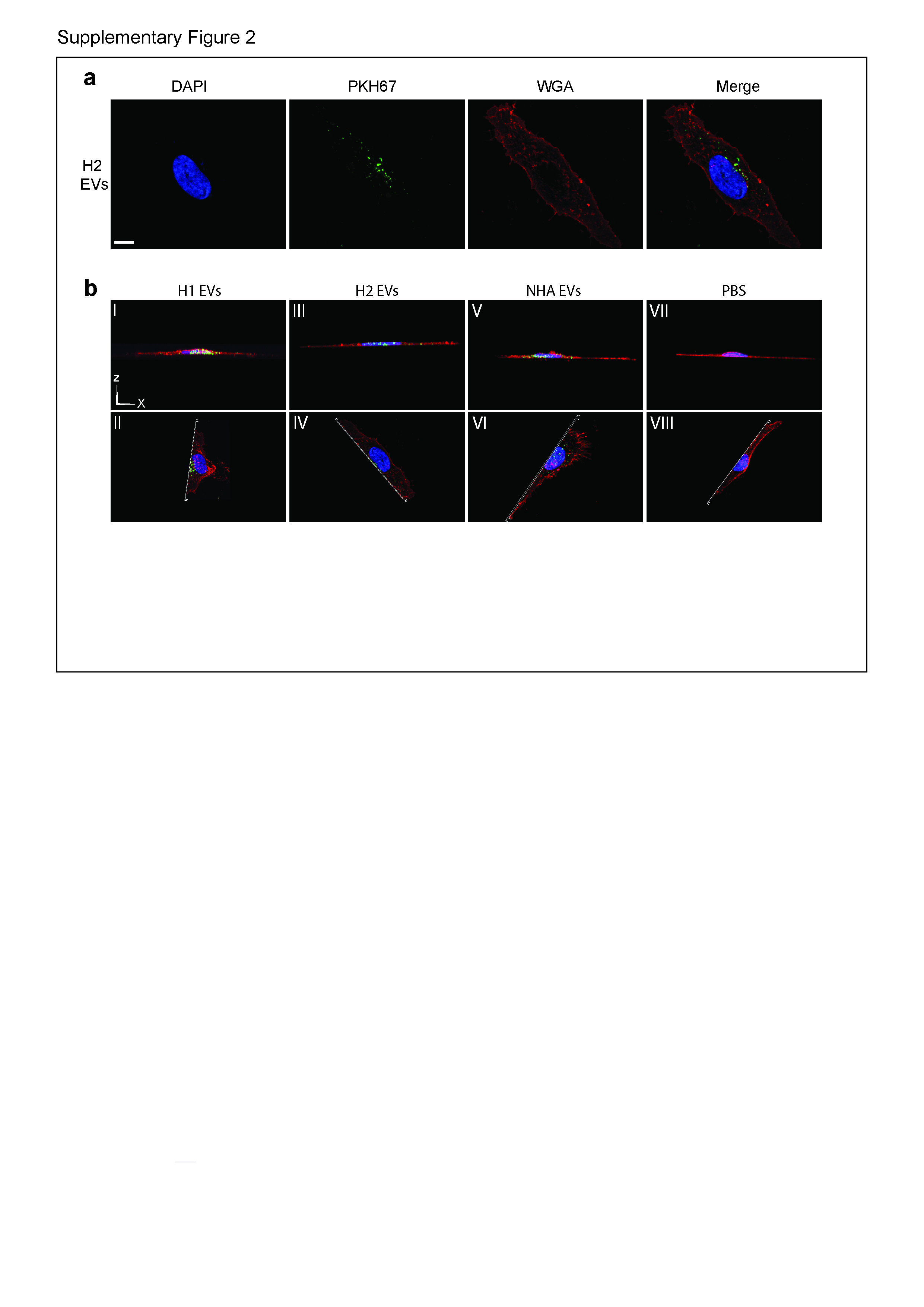

Supplement: Supplementary file 3 — Supplementary Information [file JEV2-12-12363-s005.tif]

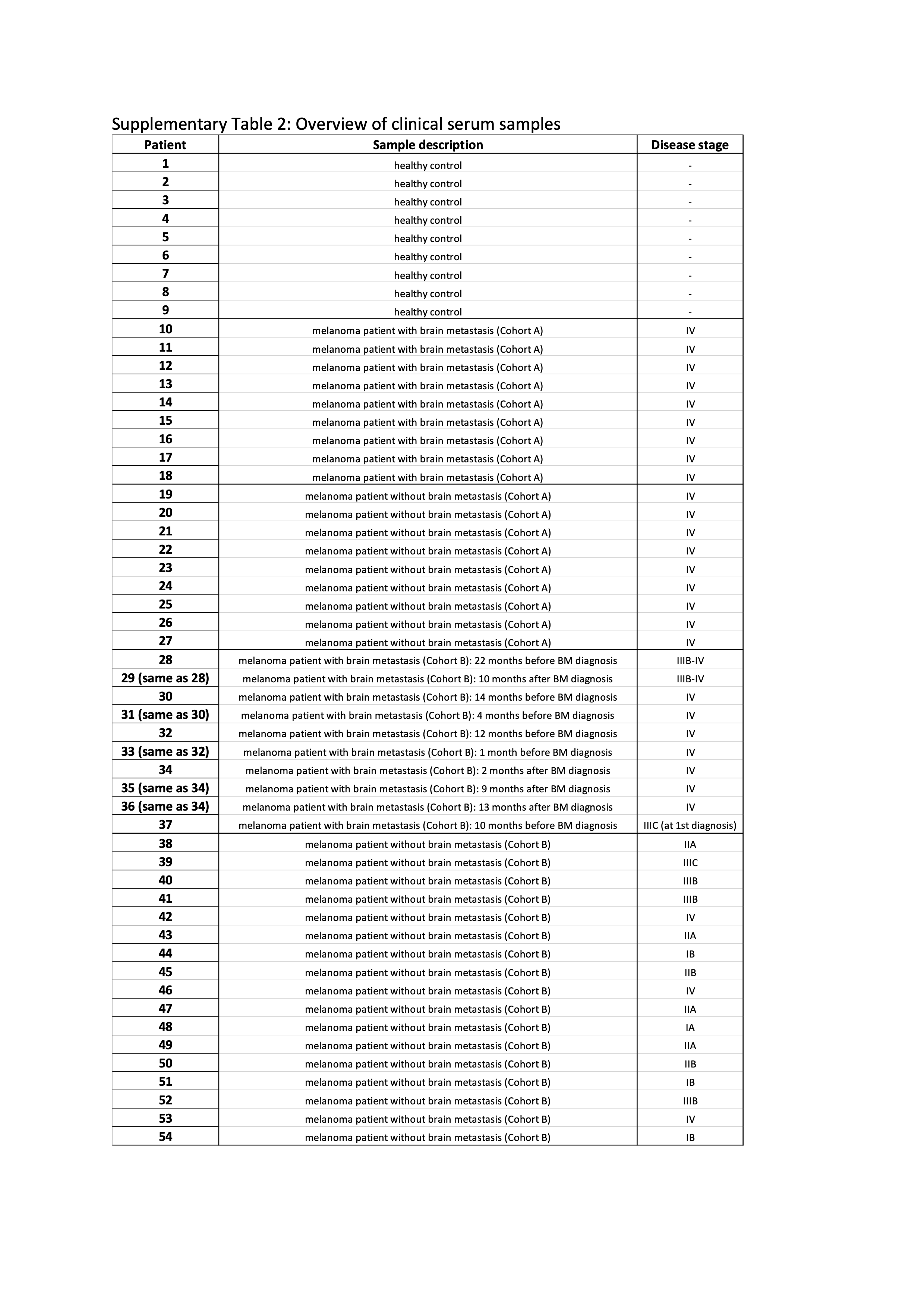

Supplement: Supplementary file 4 — Supplementary Information [file JEV2-12-12363-s009.tiff]

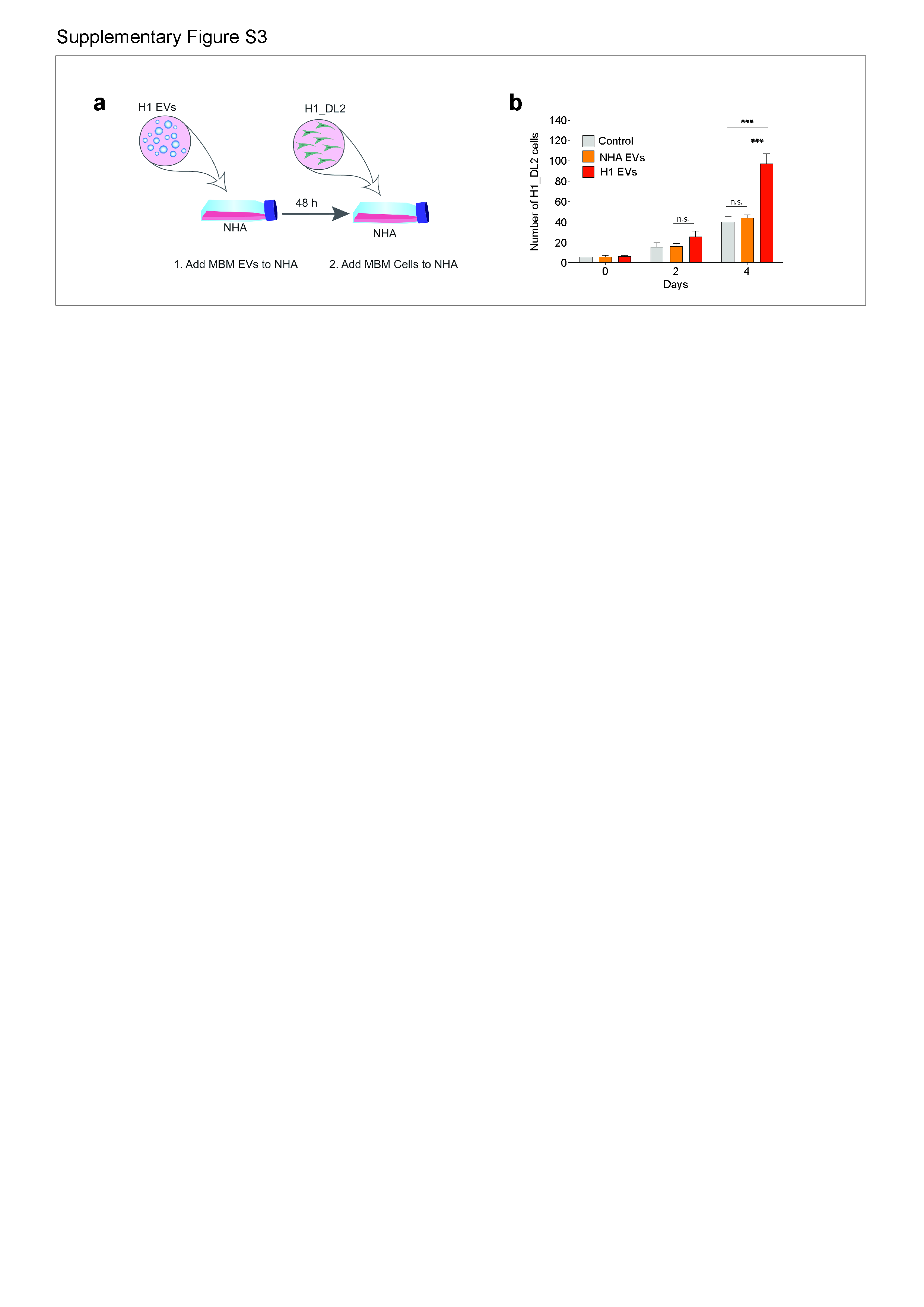

Supplement: Supplementary file 5 — Supplementary Information [file JEV2-12-12363-s003.tif]

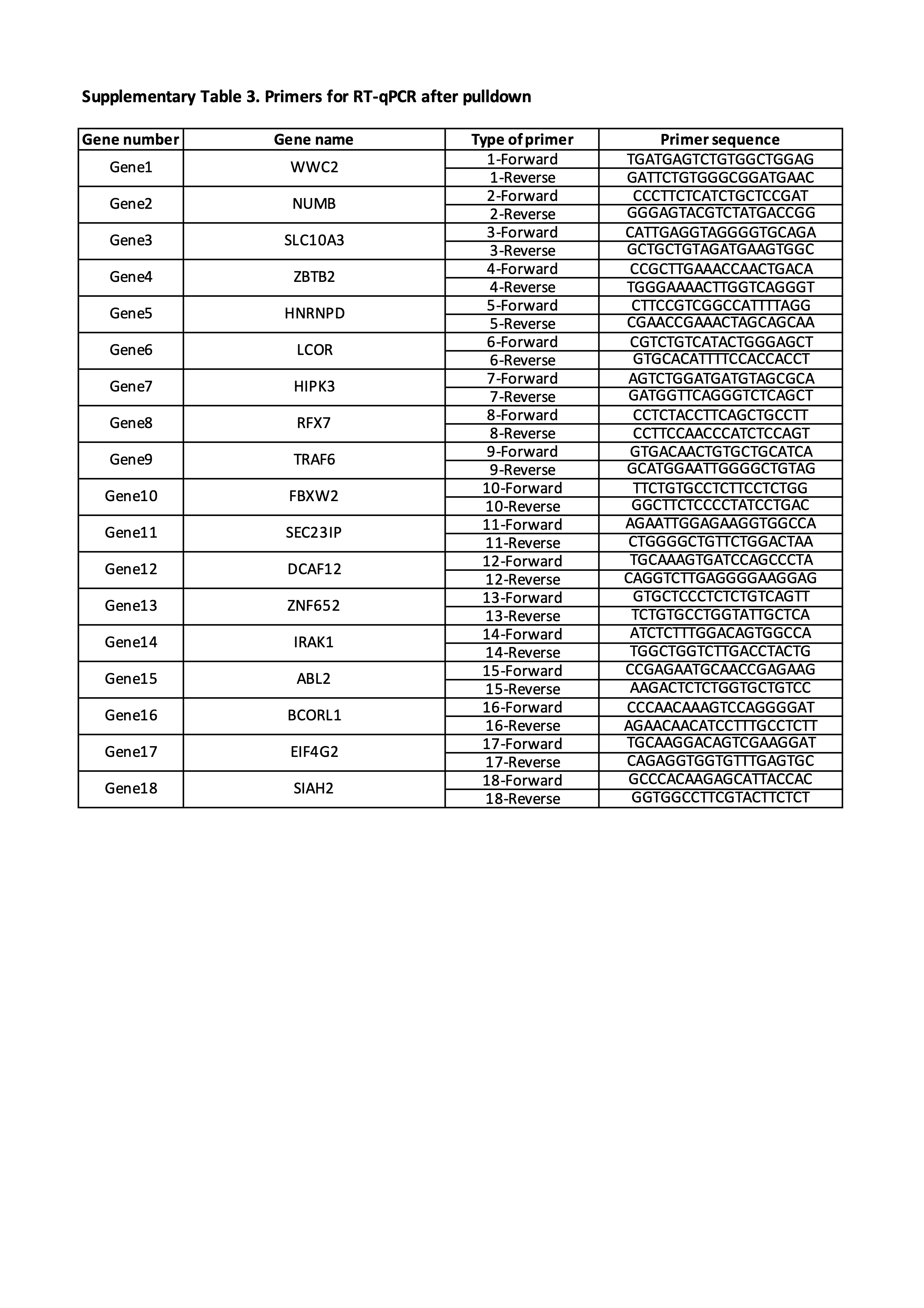

Supplement: Supplementary file 6 — Supplementary Information [file JEV2-12-12363-s006.tiff]

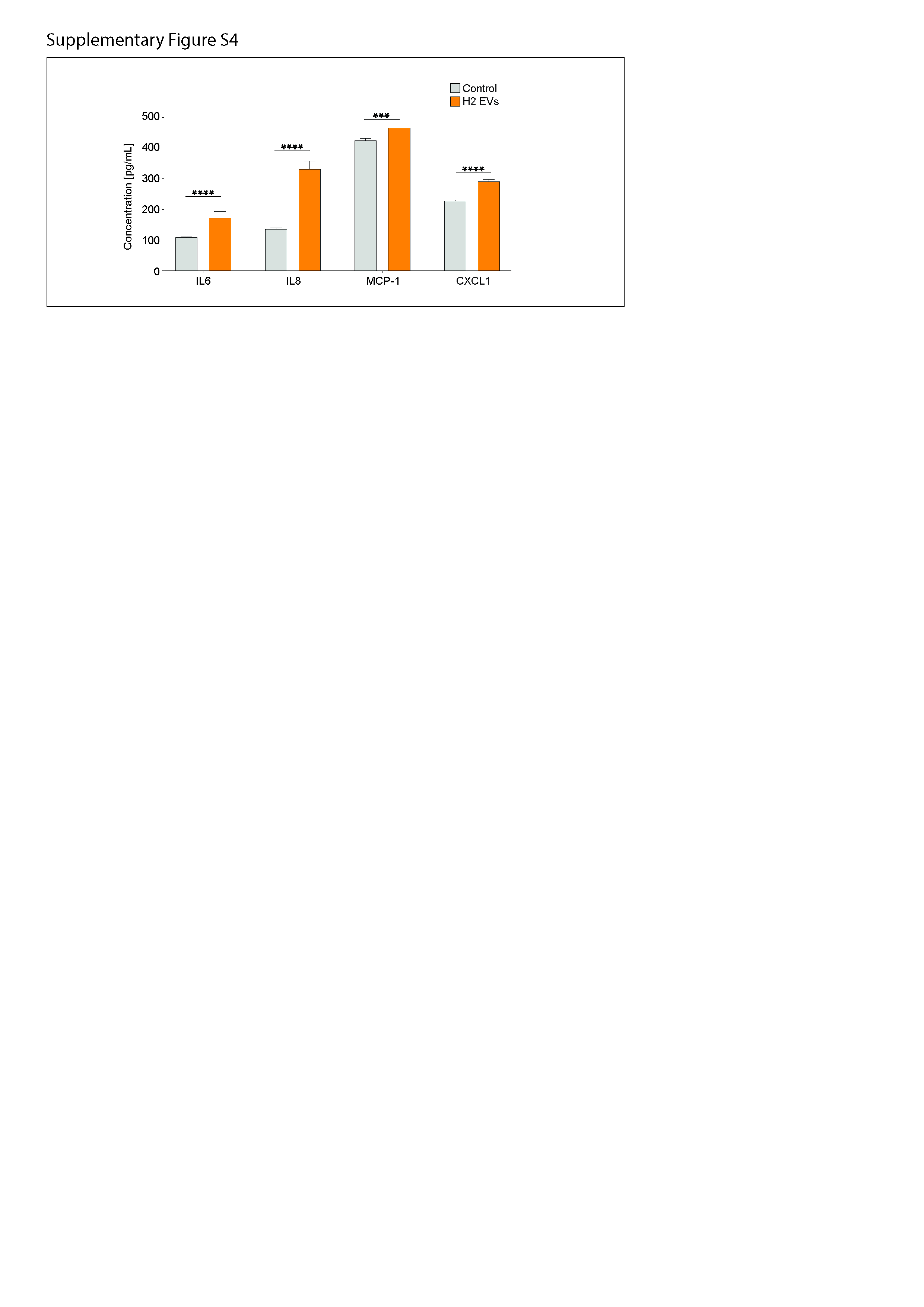

Supplement: Supplementary file 7 — Supplementary Information [file JEV2-12-12363-s008.tif]

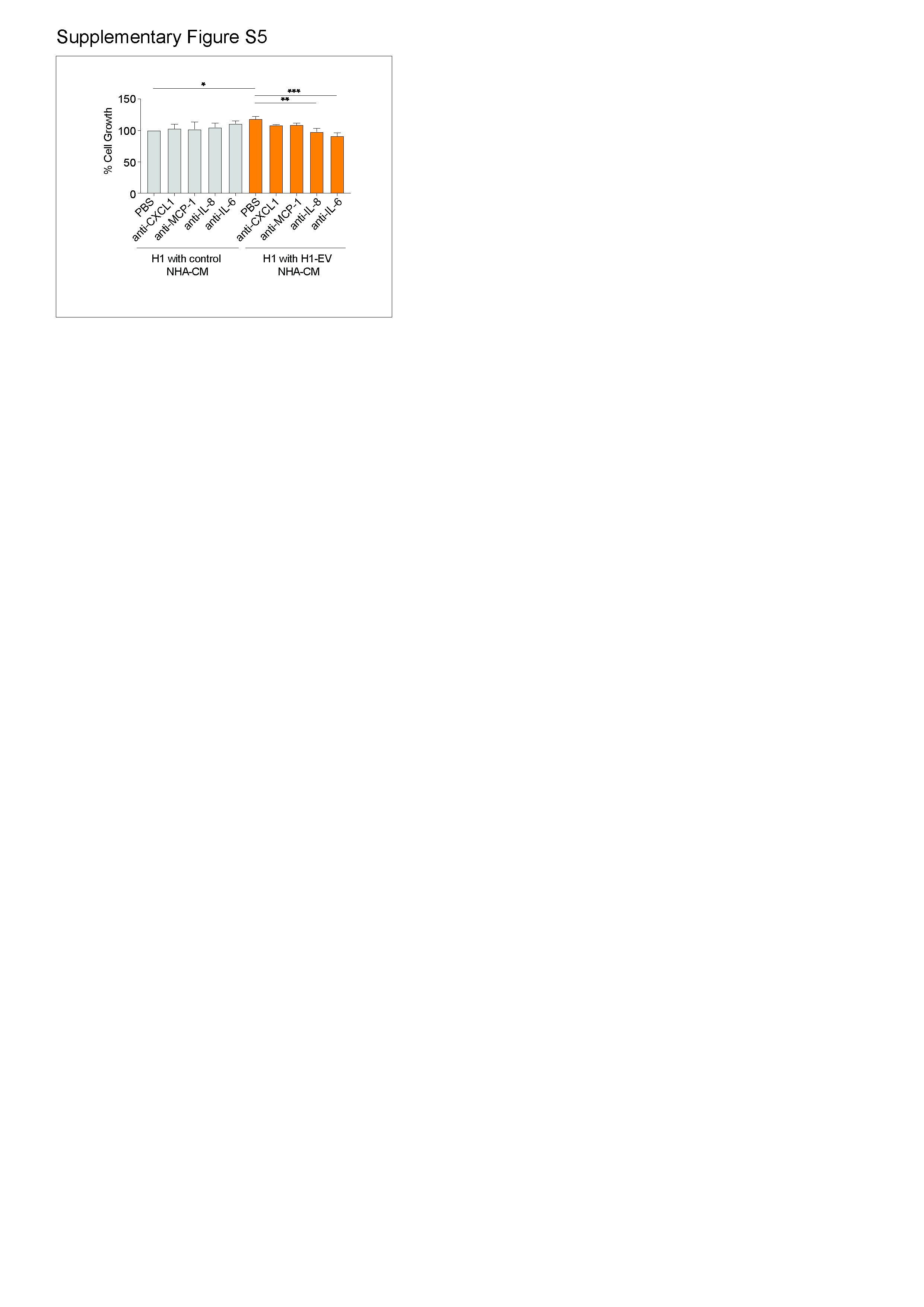

Supplement: Supplementary file 8 — Supplementary Information [file JEV2-12-12363-s015.tif]

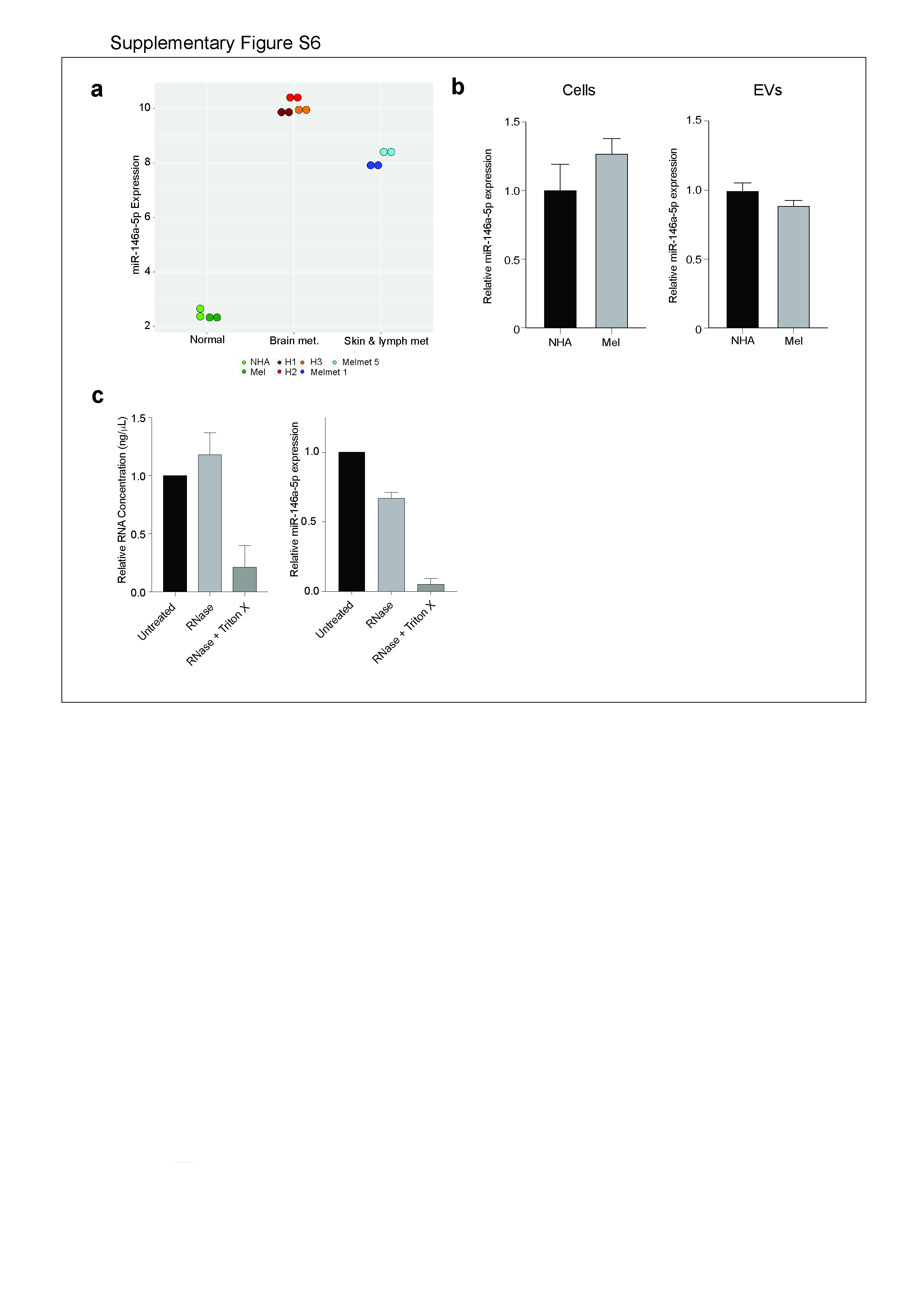

Supplement: Supplementary file 9 — Supplementary Information [file JEV2-12-12363-s019.tif]

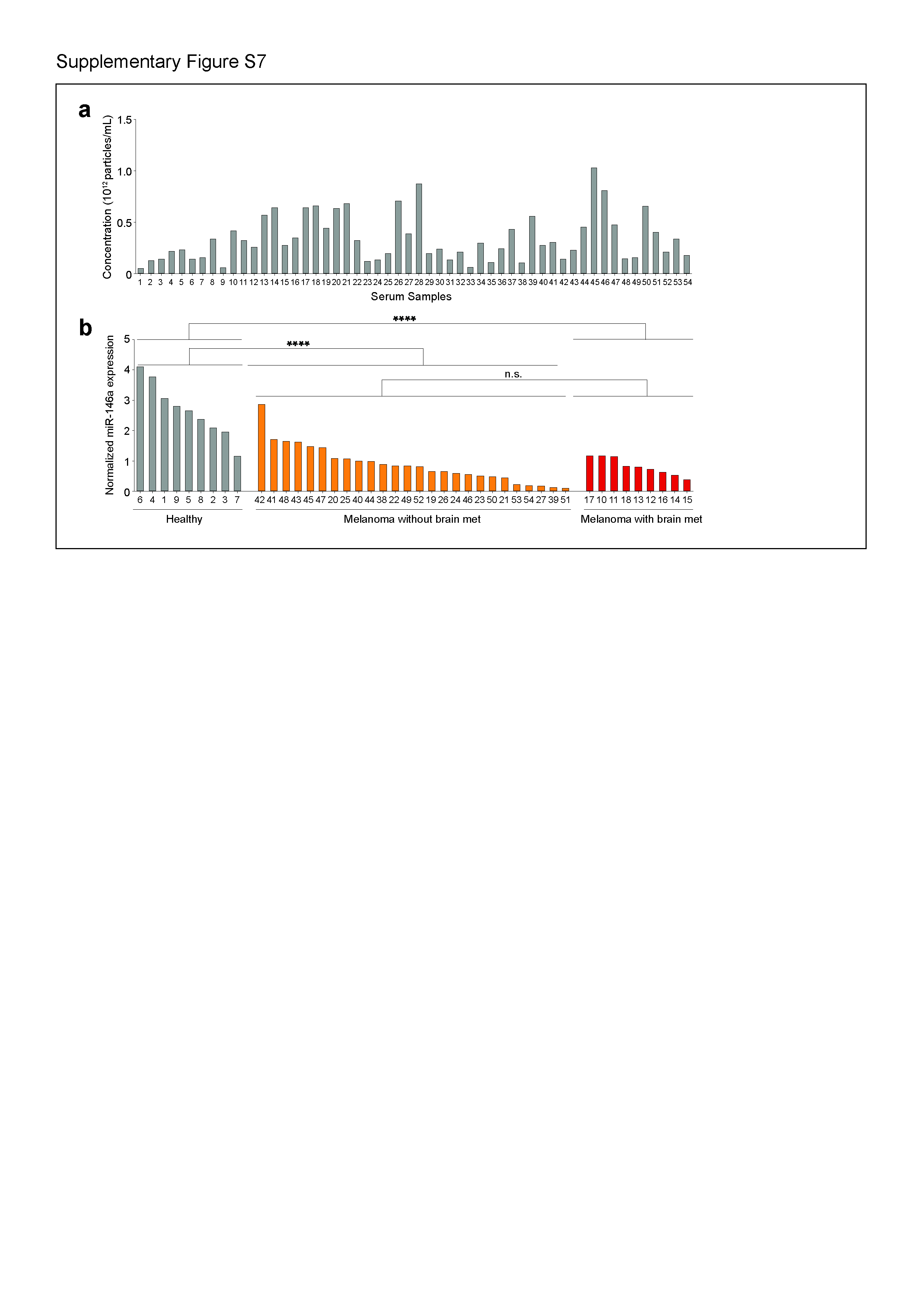

Supplement: Supplementary file 10 — Supplementary Information [file JEV2-12-12363-s010.tif]

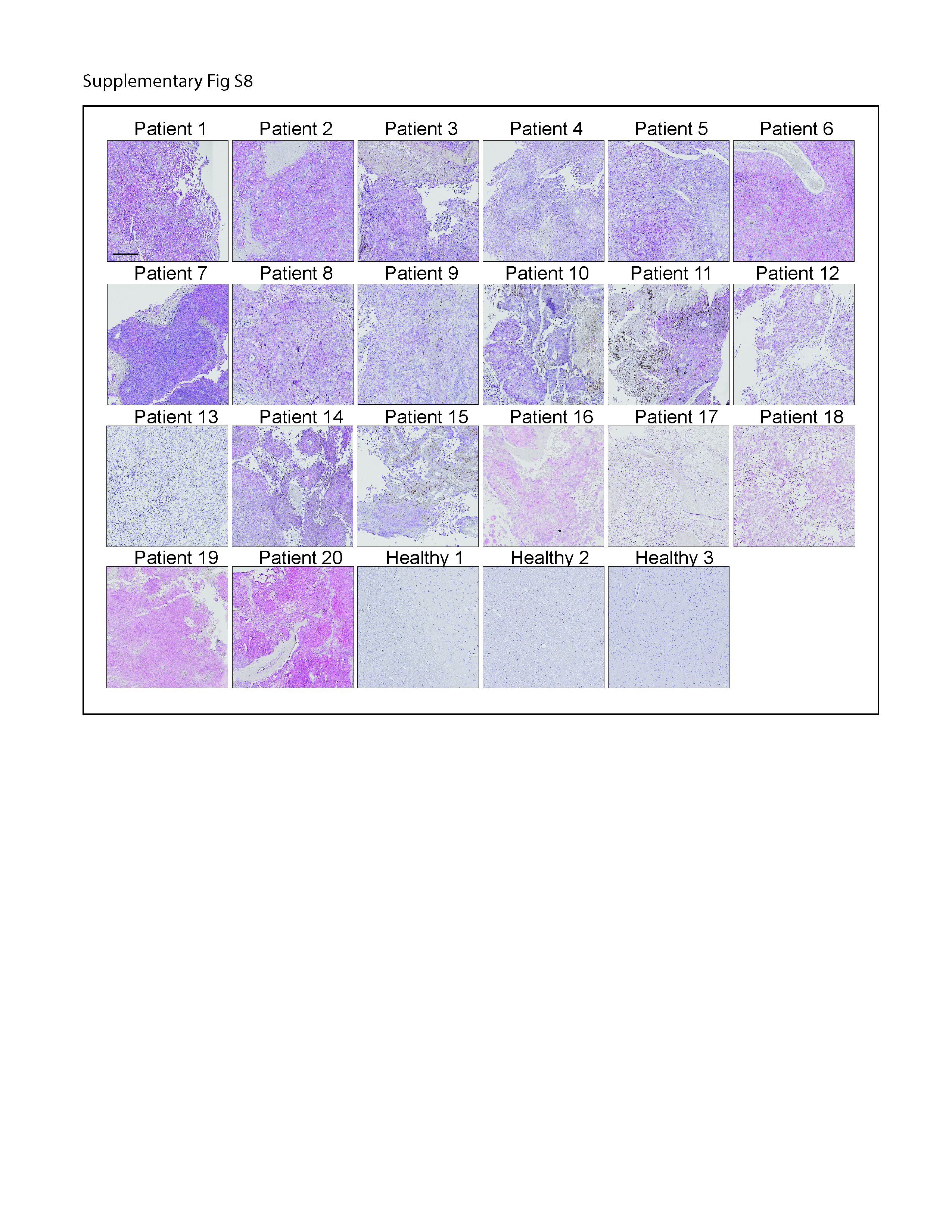

Supplement: Supplementary file 11 — Supplementary Information [file JEV2-12-12363-s002.tif]

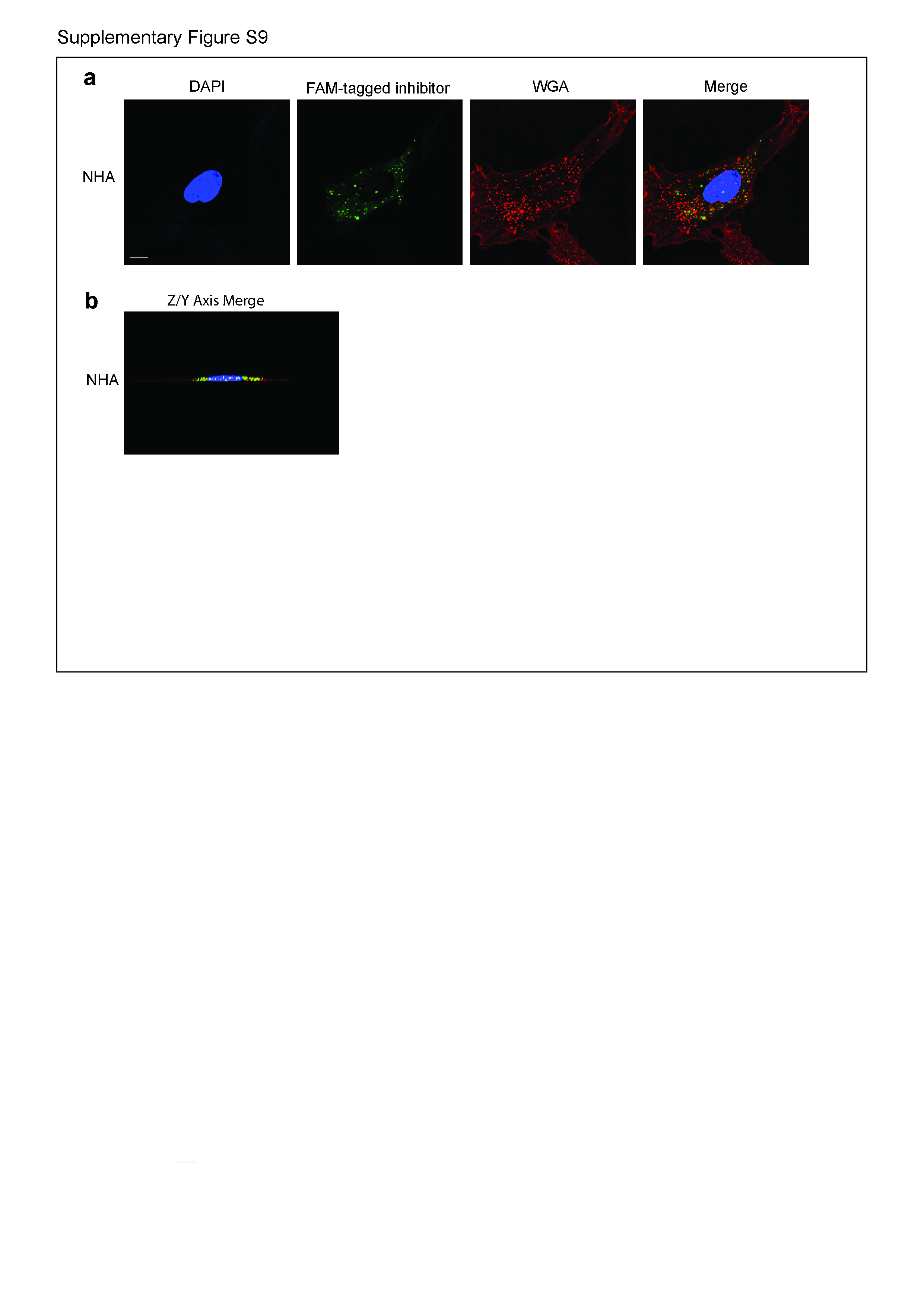

Supplement: Supplementary file 12 — Supplementary Information [file JEV2-12-12363-s016.tif]

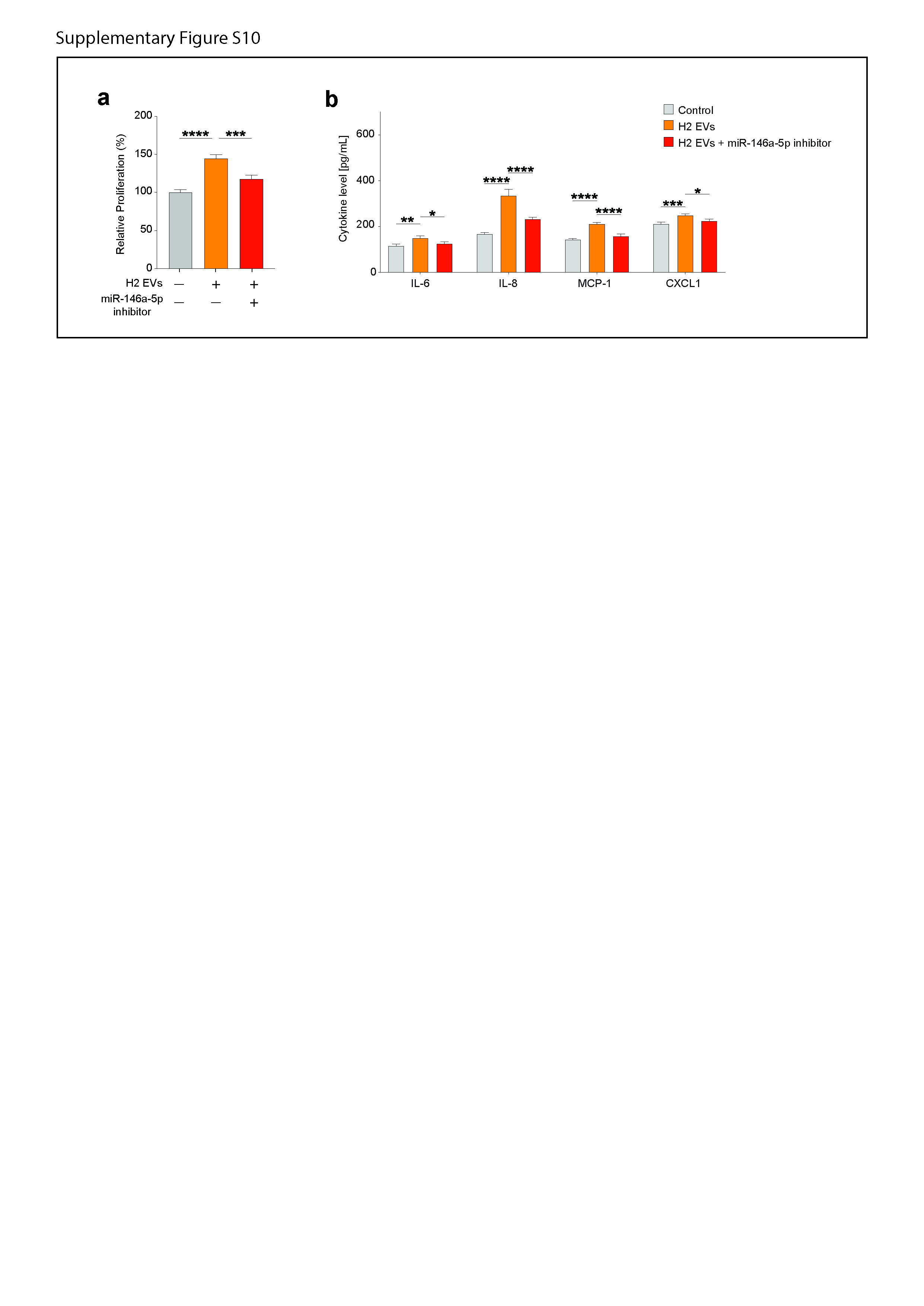

Supplement: Supplementary file 13 — Supplementary Information [file JEV2-12-12363-s020.tif]

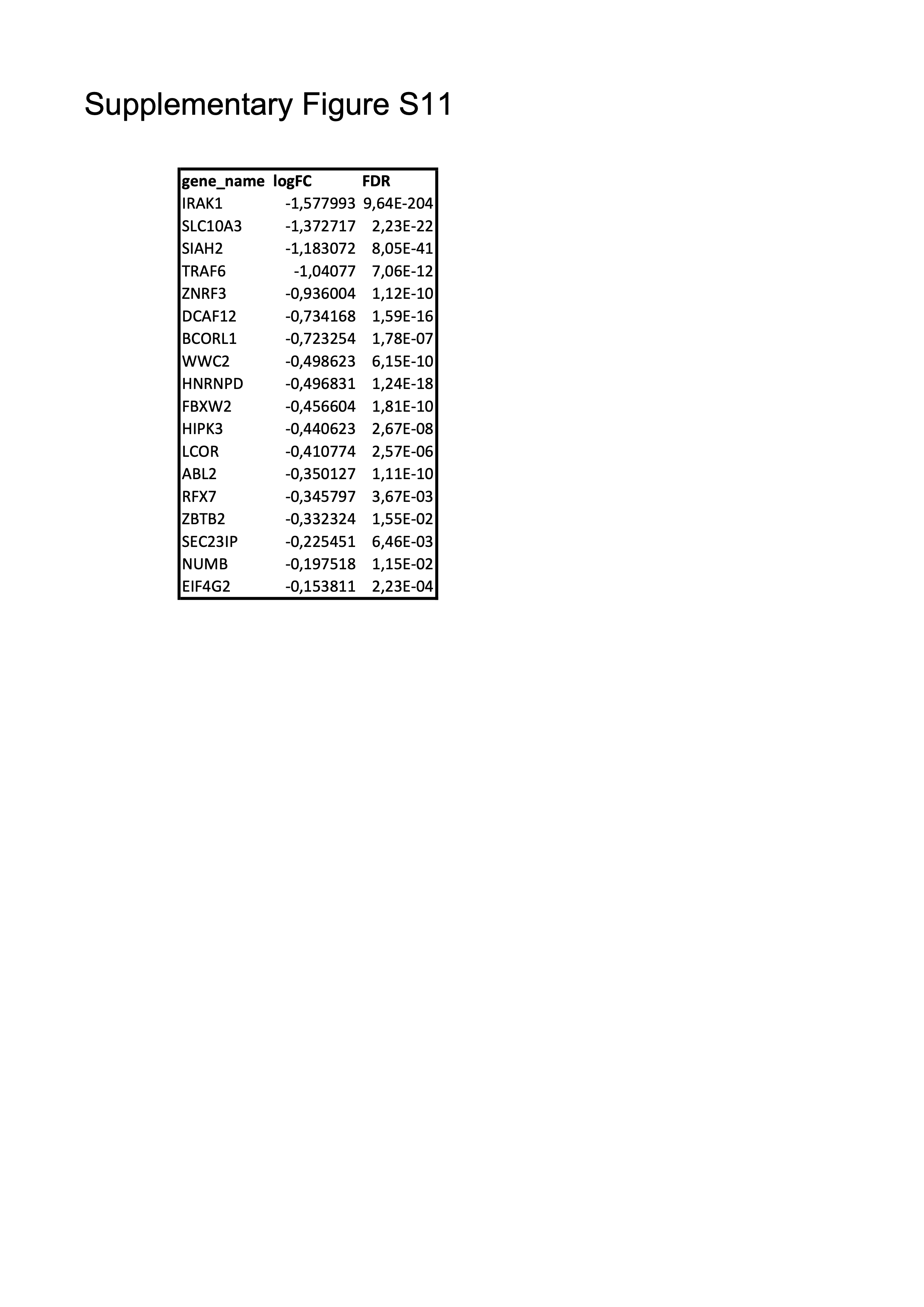

Supplement: Supplementary file 14 — Supplementary Information [file JEV2-12-12363-s018.tiff]

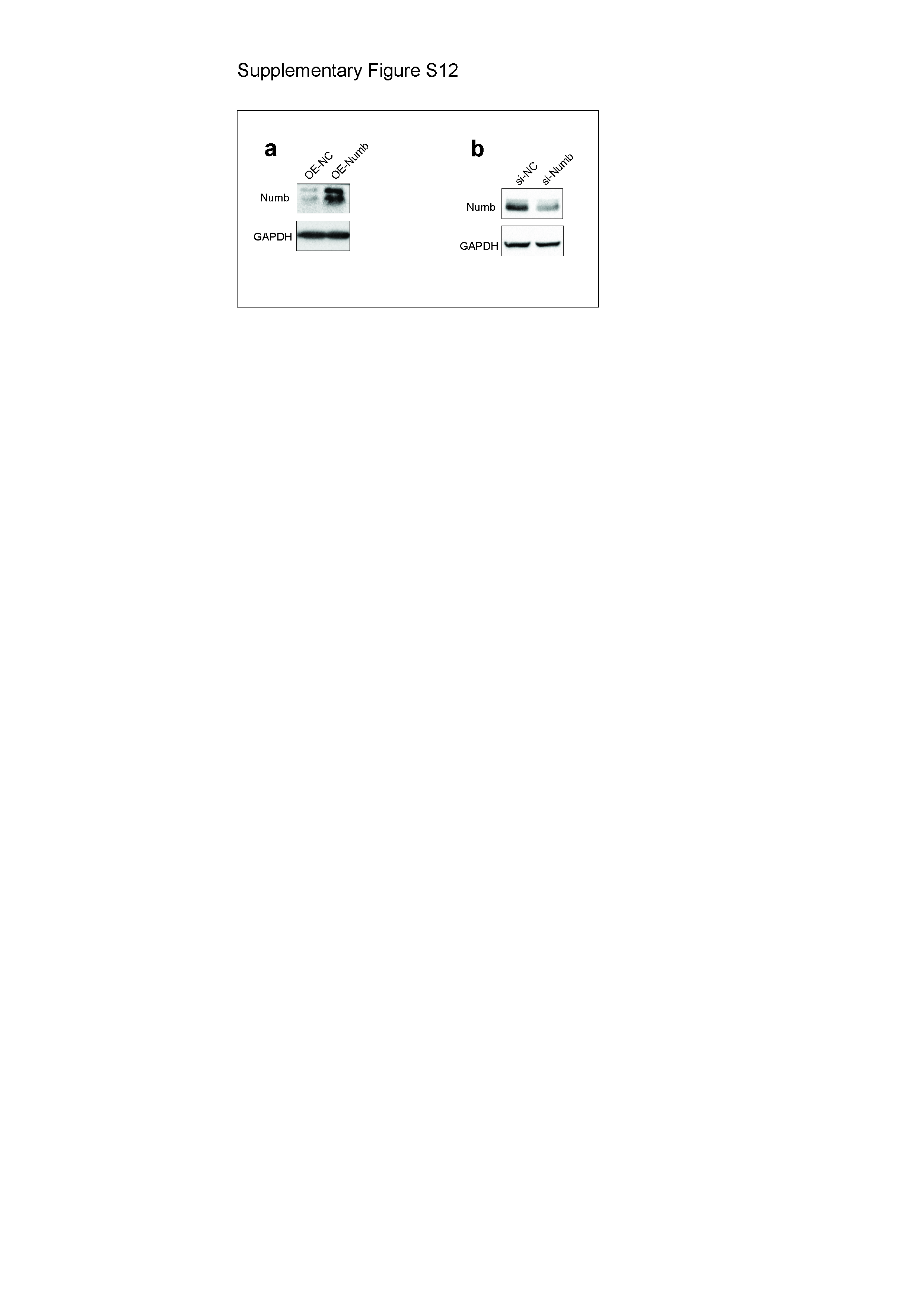

Supplement: Supplementary file 15 — Supplementary Information [file JEV2-12-12363-s014.tif]

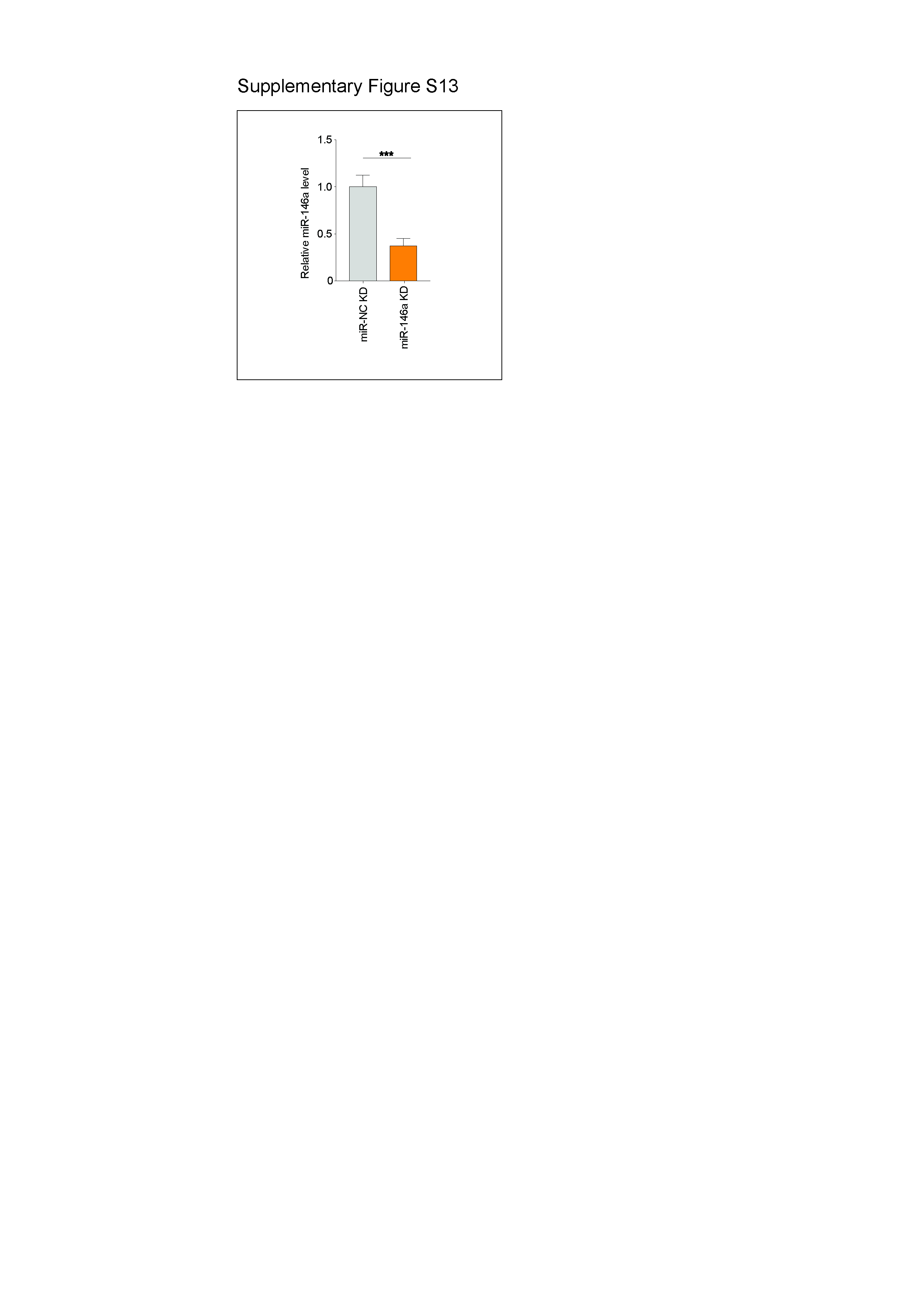

Supplement: Supplementary file 16 — Supplementary Information [file JEV2-12-12363-s011.tif]

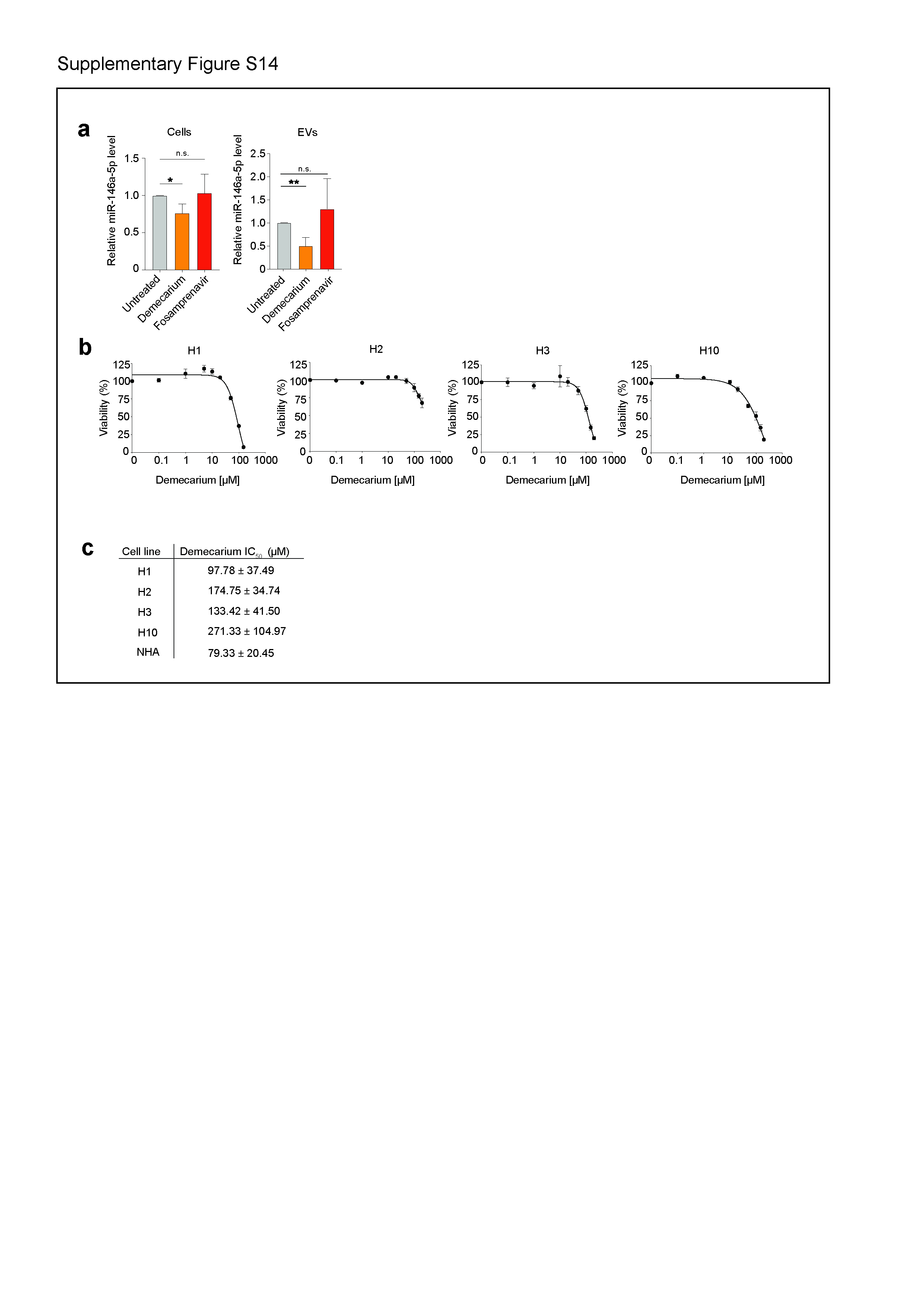

Supplement: Supplementary file 17 — Supplementary Information [file JEV2-12-12363-s001.tif]

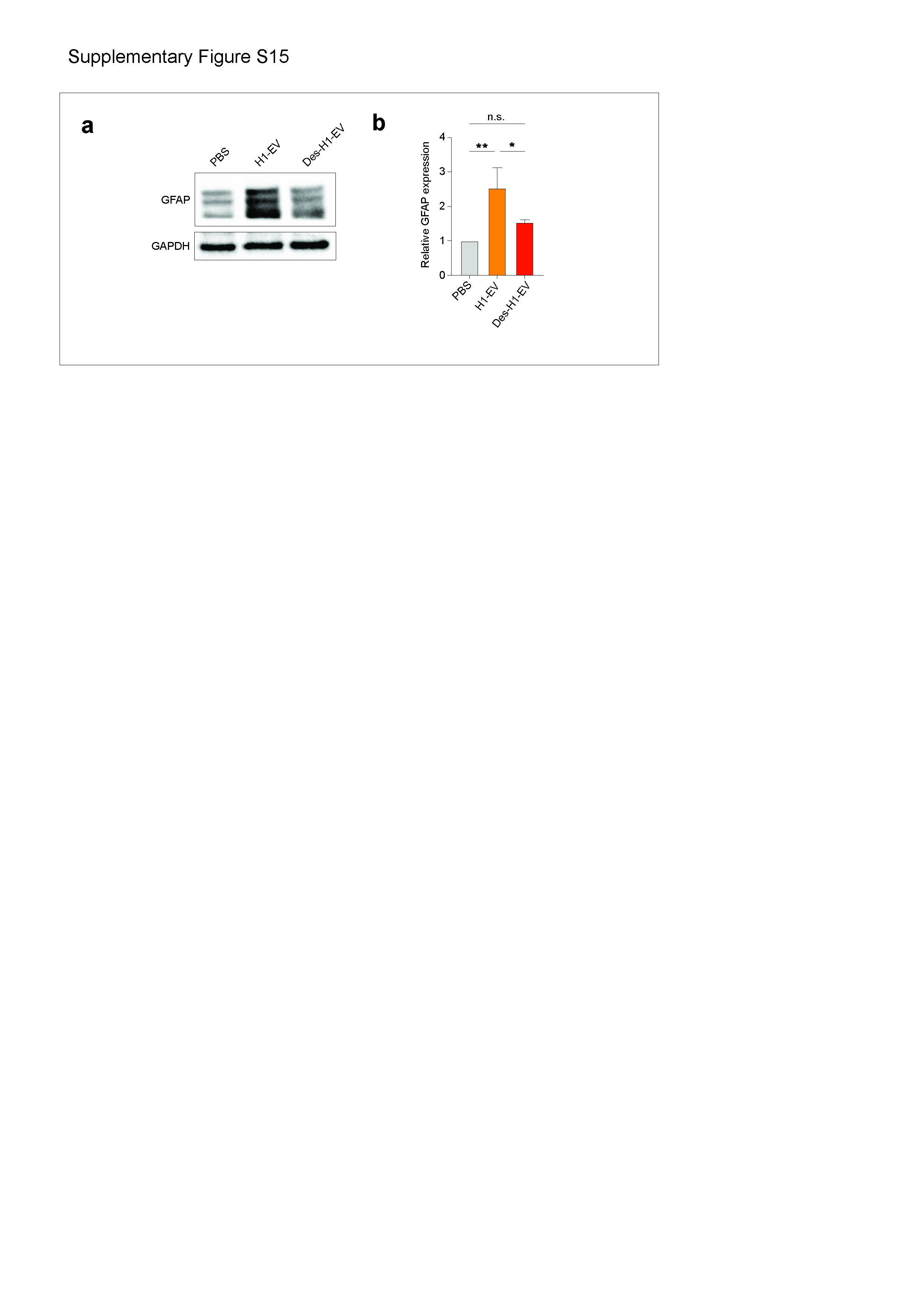

Supplement: Supplementary file 18 — Supplementary Information [file JEV2-12-12363-s004.tif]
