# Supplementary material for: Inhibition of extracellular vesicle‐derived miR‐146a‐5p decreases progression of melanoma brain metastasis via Notch pathway dysregulation in astrocytes
Source: J Extracell Vesicles. 2023 Sep 27;12(10):12363. doi: 10.1002/jev2.12363 (PMC10533779; doi:10.1002/jev2.12363)
Supplement: Supplementary file 19 — Supplementary Information [file JEV2-12-12363-s013.docx]

**Supplementary Fig. S1: Characterization of isolated EVs. A** Representative nanoparticle tracking analysis of H2-, NHA- and Mel-EVs. **B** Electron micrograph of NHA- and H2-EVs. Scale bar = 100μm. **C** Western blot analysis of EV-characteristic markers on H3, H2 and NHA cells and corresponding EVs.

**Supplementary Fig. 2: Visualization of MBM-EV uptake in NHAs. A** Confocal images of NHA cultured with 5.0 x 10^9^ EVs of PKH67-stained (green) H2-EVs for 48 h to show uptake. Nuclei stained with DAPI (blue), membrane stained with WGA-Texas Red (red). The images were obtained in X-Y orientation. Magnification (100X). Scale bar = 10 μm **B** Z-stack images of NHA cells after co-culture with PBS or H1-, H2-, and NHA-EVs to show EVs inside the plasma membrane. Images I, III, V, and VII show Z-stack in Z-X orientation. Images II, IV, VI, and VIII show corresponding images in X-Y orientation with white line indicating the slice orientation shown in the top images.

**Supplementary Fig. 3: NHA activated by H1-EVs increase MBM growth *in vitro*. A** Schematic of workflow for *in vitro* co-culture experiment. **B** Number of H1_DL2 cells at 0, 2 or 4 days after co-culture with PBS, NHA- or H1-EVs co-cultured with NHA. Cell numbers were determined by counting number of fluorescent green cells with a Nikon TE2000 inverted microscope. n.s.= not significant, ***p < 0.001.

**Supplementary Fig. 4: H2-EVs increase cytokine production in NHA cells.** ELISA validation performed on conditioned media from NHA cells co-cultured with H2-EVs. ELISA was done on the top four upregulated cytokines, IL-6, IL-8, MCP-1 and CXCL1. ***p < 0.001; ****p < 0.0001.

**Supplementary Fig. 5: Conditioned medium from NHA treated with H1-EV increases H1 growth due to cytokine upregulation.** WST-1 proliferation assay of H1 cells cultured in CM from NHA cells treated H1-EVs or PBS for 48 h. H1 cells were treated with antibodies for either CXCL1, MCP-1, IL-8 or IL-6 for 48 h. **p < 0.01, ****p < 0.0001.

**Supplementary Fig. 6: miR-146a-5p expression in normal cell- and MBM cell-EVs A** Dot plot presenting log_2_ expression of miRNA 146a in normal controls (left), brain metastases (middle) and skin and lymph node metastases (right). **B** qPCR comparing expression levels of miR-146a-5p in healthy melanocyte and NHA cells and EVs. **C** RNA protection assay of H1-EVs after treatment with RNase, RNase and Triton X compared to no treatment showing RNA concentration (left) after treatment, and qPCR of miR-146a levels (right).

**Supplementary Fig. 7: MiR-146a-5p expression levels in clinical serum EV samples.**

**A** Overall concentrations of EVs isolated from patient samples 1-54 quantified by NanoSight NS300 instrument. **B** Normalized miR-146a-5p expression in EVs from serum of healthy volunteers, melanoma patients without brain metastases, and melanoma patients with brain metastases. Data were acquired via RT qPCR analysis, results were calibrated with spike-in *C. elegans* miR-39-3p and normalized using global mean/miRNA.

**Supplementary Fig. 8: *In situ* hybridization of clinical MBM samples and normal brain tissue.** miRNAscope *in situ* hybridization assay of miR-146a-5p expression in patient MBM samples and healthy brain controls. Red dots indicate successful binding of the miR-146a probe, tissues counterstained with hematoxylin (purple). Scale bar = 200μm.

**Supplementary Fig. 9: Visualization of FAM-tagged miR-146a inhibitor in NHA cells.**

Confocal images of NHA uptake of FAM-tagged miR-146a inhibitor (green) after 24 h. Nuclei stained with DAPI (blue), membrane stained with WGA-Texas Red (red). The images were obtained in X-Y orientation. Magnification (100X). Scale bar = 10 μm **B** Z-stack image of NHA cells after addition of inhibitor to confirm presence inside the plasma membrane in Z-Y orientation.

**Supplementary Fig. 10: A** CCK8 proliferation assay of NHA cells after co-culture with 5.0 x 10^9^ H2-EVs in the presence or absence of a miR-146a-5p inhibitor. **B** ELISA of IL-6, IL-8, MCP-1 and CXCL1 levels from NHA cell conditioned media after co-culture with 5.0 x 10^9^ H2-derived EVs in the presence or absence of miR-146a-5p inhibitor. *p < 0.05, **p < 0.01, ***p < 0.001; ****p < 0.0001.

**Supplementary Fig. 11: 18 common predicted miR-146a-5p binding partners.** Log fold change (logFC) and false discovery rate (FDR) of 18 predicted binding partners of miR-146a-5p from three online databases (TargetScan, miRDB, and microT-CDS) combined with sequencing data in Figure 4a.

**Supplementary Fig. 12: Overexpression and silencing of NUMB in NHA cells. A** Western blot confirming NUMB overexpression in NHA cells. Overexpression performed by transfection of an overexpression plasmid vector. **B** Western blot confirming NUMB silencing. Silencing performed by addition of NUMB-siRNA treatment of NHA cells.

**Supplementary Fig. 13: A** **MiR-146a-5p is knocked down in H1_DL2 cells.** qPCR confirming successful knockdown of miR-146-5p in H1_DL2 cells via transfection of a miRNA sponge lentiviral vector. ***p < 0.001.

**Supplementary Fig. 14: Screening of drug candidates demecarium bromide and fosamprenavir. A** qPCR analysis of miR-146a-5p expression in H1 cells and H1 EVs after demecarium bromide and fosamprenavir treatment. **B and C** Representative IC_50_ survival curves of H1, H2, H3 and H10 cells after treatment with increasing demecarium bromide concentrations (0.1 µM–1000 µM) for 72 h and corresponding IC_50_ doses of all MBM cell lines and NHA. n.s.= not significant *p < 0.05, **p < 0.01.

**Supplementary Fig. 15**: **EVs derived from deserpidine-treated H1 cells do not activate NHA.** **A** Western blot of NHA cells after 48 h co-culture with either PBS, H1-EV or 20 µM deserpidine treated H1-EV (Des-H1-EV). **B** Subsequent quantification of western blot normalized to PBS control. n.s.= not significant *p < 0.05, **p < 0.01.
