# Supplementary material for: Inhibition of extracellular vesicle‐derived miR‐146a‐5p decreases progression of melanoma brain metastasis via Notch pathway dysregulation in astrocytes
Source: J Extracell Vesicles. 2023 Sep 27;12(10):12363. doi: 10.1002/jev2.12363 (PMC10533779; doi:10.1002/jev2.12363)
Supplement: Supplementary file 20 — Supplementary Information [file JEV2-12-12363-s007.docx]

**Supplementary Materials and Methods**

**2.3 Western blotting (WB)**

Cells and EV lysates were isolated with RIPA lysis buffer supplemented with 10X protease and phosphatase inhibitor cocktail (Roche, Basel, Switzerland). Protein concentrations were measured using a Pierce BCA assay (Life Technologies, San Diego, CA, USA). Equal amounts of protein were diluted in NuPAGE™ 4X LDS Sample Buffer (Invitrogen, Waltham, MA, USA) and 10X Sample Reducing Agent (Invitrogen) and electrophoresed on a 10% or 12% Tris-Glycine SDS-polyacrylamide gel. Gels were run at 80V for stacking and 120V for separation until sample dye reached the bottom of the gel. Samples were transferred to a nitrocellulose membrane at 100 V for 1 h. Membranes were blocked for 1 h at room temperature (RT) with 5% skim milk in 1X TBS-T solution and incubated with primary immunoblotting antibodies: GAPDH (cat. no. 5174; Cell Signaling Technology, Inc., Danvers, MA, USA), GFAP (cat. no. 80788; Cell Signaling Technology), NUMB (cat. no. 2756; Cell Signaling Technology), CD9 (cat. no. 13118; Santa Cruz Biotechnology), Flotillin-1 (cat. no. 74566; Santa Cruz Biotechnology), TSG-101 (cat. no. 7964; Santa Cruz Biotechnology), Calnexin (cat. no. 46669; Santa Cruz Biotechnology) overnight at 4^0^C. Membranes were washed and incubated with secondary antibodies (HRP anti-rabbit IgG; HRP 1:10,000, anti-mouse IgG 1:10 000; Santa Cruz Biotechnology, Inc, Dallas, TX, USA) for 2 h at room temperature. Signals were visualized with SuperSignal™ Pico/Femto Chemiluminescent Substrate (Thermo Fisher Scientific, Waltham, MA, USA) using a LAS-3000 imaging system (Fujifilm, Saitama, Japan).

**2.4 Animals**

Female NOD/SCID mice or female nude mice (6-8 weeks old) were fed a standard pellet diet and provided water *ad libitum*. Anesthesia was induced with 3% isoflurane (Abbott Laboratories, Chicago, IL, USA) in oxygen and maintained with 1.5% isoflurane in oxygen during all procedures unless stated otherwise. The mice were monitored daily and sacrificed when significant morbidity symptoms were observed. The Norwegian National Animal Research Authority (Application #14751, approved Feb 6^th^, 2018, and Application #28740, approved Dec 21^st^, 2021) and The Institutional Animal Care and Use Committee (IACUC) of Shandong University, Jinan, China (Approval number: KYLL-2020(KS)-515) approved all animal procedures prior to all experiments.

**2.5 Visualization of EVs *in vivo***

EVs and Cy7 NHS ester (cat. no. A8109; APExBIO, Houston, TX, USA) were mixed at a mass ratio of 100:1 and incubated at 37^0^C for 15 min in the dark. Cy7-stained EVs and unstained EVs (as negative control) were then transferred to 300 kDa Vivaspin centrifugal concentrators (Sigma-Aldrich, St. Louis, MO, USA). The samples were centrifuged at 4,000*g* and checked in between to ensure that a small part of the sample remained on top of the filter. Samples were washed with 5 ml PBS and the centrifugation procedure with subsequent washing step was repeated.

5 x 10^9^ Cy7-labeled EVs in 100 μL PBS was injected intracardially into a nude mouse. Mice were fluorescently imaged after 24 h using the IVIS-200 optical imaging (OI) system (Xenogen, Alameda, CA, USA). The mice were then sacrificed by perfusion of 0.9% saline solution into the left cardiac ventricle. Brains, hearts, lungs, livers, spleens, kidneys, and intestines were removed, and *ex vivo* fluorescence imaging was done on the organs, using the IVIS-200 OI system (Xenogen, Alameda, CA, USA).

- 1. **Assessment of H1_DL2 growth cocultured with activated NHA**

NHA cells were seeded at 5.0 x 10^3^ cells/well in 200 μL into each well of a 96 well dish (Nunc) and left for 24 h. Then, NHA were incubated with 5.0 x 10^9^ EVs/mL from H1 or NHA cells or with PBS for 48 h, before being cocultured with H1_DL2 cells. Proliferation of H1_DL2 cells during 96 h was assessed *in vitro* using a Nikon TE2000 inverted microscope (Nikon Instruments Inc., Melville, NY. USA).

**2.7 Assessment of MBM burden in mice after intracardial pre-injections of EVs**

12 female NOD/SCID mice were anesthetized with 3% sevoflurane in oxygen and anesthesia was maintained with 1.5% sevoflurane in oxygen. The mice were fixed in a supine position on a heating pad to maintain a core temperature of 37^0^C. The mice were randomized into two groups (6 mice per group). One group received 3 injections (once/day) with 5.0 x 10^9^ EVs from H1 MBM cells in 100 μL PBS (in total 1.5 x 10^10^ EVs), while the second group received 3 injections (once/day) of 100 μL PBS. All injections were done intracardially, using a 30G insulin syringe (Omnican50, B. Brain Medical AS, Vestskogen, Norway), by ultrasound guidance (Vevo 2100 Imaging System, VisualSonics Inc., Toronto, Canada). After 2 days, 5.0 x 10^5^ H1_DL2 MBM cells in 100 μL PBS were injected intracardially into all mice, by ultrasound guidance.

- 1. ***In vivo* magnetic resonance imaging (MRI)**

Brain metastasis development in the NOD/SCID mice after intracardiac injections of EVs and tumor cells was studied at weeks 4 and 6 using a 7 Tesla Pharmascan small-animal MR scanner (Bruker BioSpin MRI, Ettlingen, Germany) equipped with a 1-channel circular transmitter coil and a 4-channel receiver surface coil. T2 weighted (T2w) coronal images were obtained with the following RARE sequence scan parameters: FOV 20 mm × 20 mm, matrix size 256 × 256, 0.5 mm slice thickness, TR 3200 ms, TE 38 ms, FA 90^0^, 4 averages, scan time 6 min 49 s.

Brain metastasis development in the NOD/SCID mice after intracardiac injections of tumor cells followed by deserpidine treatment was studied at weeks 4 and 6 using a 7 Tesla small-animal PET/MR scanner (MR Solutions Ltd., Guildford, UK) equipped with a 20mm inner diameter quadrature transmit/receive volume coil. T2w coronal images were obtained using the following fast spin echo sequence scan parameters: FOV 20 mm × 20 mm, matrix size 256 × 256, 0.5 mm slice thickness, TR 3000 ms, TE 45 ms, FA 90^0^, 2 averages, scan time 3 min 41 s.

Visualization of MR images and quantification of tumor numbers and volumes (V = 4/3 × ∏ × r^3^) were performed using the 32-bit OsiriX freeware, version 5.8.1 (Pixmeo SARL, Geneva, Switzerland) in both studies.

**2.9 *In vitro* uptake of EVs/miRNA inhibitors into normal human astrocytes (NHA)**

Confocal imaging was used to visualize and confirm the uptake of either EVs or miRNA inhibitors into NHA cells.

For confirmation of uptake of EVs into NHA cells, EVs from H1, H2 and NHA cells were labeled using the PKH67 Green Fluorescent Cell Linker Kit (Sigma-Aldrich), as follows: 5.0 x 10^9^ EVs, or equivalent volume PBS as control, were diluted up to 1mL in Diluent C and mixed with equal volume 4.0 x 10^-6^M PKH67 dye for 5 min. Staining was quenched with 2 mL of 1% BSA solution for 1 min. Excess stain was removed by spinning samples in Vivaspin 300 kDa centrifugal filters (Sigma-Aldrich) at 4,000*g* and rinsed with 5 mL PBS 3 times. Final EV samples were diluted in growth medium and 5.0 x 10^9^ EVs/mL of each were added to 2.0 x 10^4^ NHA cells on coverslips in a 24-well plate.

To confirm the entry of miRNA inhibitors into NHA cells, a fluorescein-tagged fluorescent miR-146a-5p inhibitor (Qiagen, Düsseldorf, Germany) was added to 5.0 x 10^4^ NHA cells on coverslips in a 24-well plate.

After 24 h, NHA cells were fixed with 4% PFA for 10 min at RT and stained sequentially with 5 µg/L Wheat Germ Agglutinin (WGA), Texas Red™-X Conjugate (Invitrogen) for 10 min and 300 nM DAPI (Invitrogen) for 5 min. Cells were rinsed and mounted on slides with ProLong™ Gold Antifade Mountant (Invitrogen). Fluorescent images were obtained by confocal laser scanning microscopy using a Leica TCS SP8 system (Leica Microsystems, Wetzlar, Germany) with a 100x NA1.4 HC PL APO STED White objective. Z-stacks were captured with a step size of 0.4 μm and presented as maximum intensity projections. The experiments were done in duplicate.

**2.10 Immunofluorescence of GFAP expression**

After co-culture with 3.0 x 10^8^ EVs/mL for 48 h, NHA cells were either lysed for western blotting or were fixed with 3% paraformaldehyde (PFA) for 10 min at RT and permeabilized with 0.1% Triton X-100 for 10 min. After fixation, cells were incubated with 3% bovine serum albumin (BSA; Sigma-Aldrich) in PBS for 1 h to block nonspecific binding. NHA cells were then incubated with rabbit polyclonal antibody against GFAP (1:200, cat. no. 80788; Cell Signaling Technology) at 37^0^C for 1 h. After washing with PBS containing Mg2+ and Ca2+, the cells were incubated with Alexa Fluor 594-conjugated anti-rabbit IgG (Invitrogen) for 1 h at 37^0^C. Cell nuclei were then stained with DAPI (Thermo Fisher Scientific). Fluorescent images were obtained by confocal laser scanning microscopy using a Leica TCS SP8 system (Leica Microsystems).

**2.11 Cell proliferation and viability**

The Cell Counting Kit–8 (Sigma Aldrich 96992) was used to examine the effects of EVs from NHA and H1 on NHA, as well as to determine the IC_50_ doses of deserpidine, demecarium bromide and fosamprenavir on MBM cell lines. Drugs were all purchased from MedChemExpress, diluted in DMSO at recommended stock concentration according to the manufacturer and kept at -80^o^C for long term storage.

H1, H2, H3, H10 and NHA cells were seeded at 5.0 x 10^3^ cells/well in 200μl in 96-well plates. After 24 h, cells were treated with 5.0 x 10^9^ EVs/mL from NHA or H1 for 48 h, or deserpidine, demecarium or fosamprenavir (0, 0.1, 1, 5, 10, 20, 50, 100, and 150 µM) for 72 h. 10 µL of CCK-8 reagent was added to each well, and the plate was incubated for 4 h at 37^0^C. Absorbance of plates was measured at 450 nm using a Multiskan FC Microplate Photometer (Thermo Fisher Scientific). IC_50_ doses were calculated using GraphPad Prism v7 software (GraphPad Software Inc., La Jolla, CA, USA). The experiments were done in triplicate.

**2.12 Wound healing assays**

Migration of cells was evaluated by two different wound healing assays.

First, NHA or H1 cells were seeded at 3.0 x 10^4^ cells/well in a 96-well ImageLock plate (Essen BioScience Ltd., Welwyn Garden City, UK) and incubated for 48 h until confluency. A wound-maker tool (Essen BioScience Ltd.) was used to simultaneously create a uniform wound across all wells, after which H1 or NHA EVs, H1 EVs+miR-146a-5p inhibitor, H1 EVs+miR-146a-5p NC inhibitor or deserpidine (0, 10, 20, 30 µM) was added to the cells. The plates were imaged every 2 h for 72 h using the IncuCyte Live Cell Imaging System (Essen BioScience Ltd.), and subsequent analysis was done with the IncuCyte Scratch Wound Cell Migration Software Module (cat. no. 9600-0012; Essen BioScience Ltd.). The experiments were done in triplicate.

Second, NHA cells were seeded at 2.0 x 10^5^ cells/well in 6-well, flat-bottomed plates and incubated at 37^0^C overnight. A cell-free gap was generated by scratching with a 200 μL pipette tip. PBS or 5.0 x 10^9^ EVs/mL from NHA or H1 cells were then added to the wells. The wound closure area was measured at 0 h, 24 h, 48 h, 72 h and 96 h using a Leica DMi8 microscope (Leica Microsystems) and quantified using ImageJ v1.53c freeware (National Institutes of Health, Bethesda, MD, USA). The experiments were done in triplicate.

**2.13 Cytokine array and enzyme-linked immunosorbent assay (ELISA)**

A human cytokine antibody array (ab133996; Abcam, Cambridge, UK) was used to screen for the secretion of 23 different cytokines in the supernatant of PBS or H1 EV treated NHA cells *in vitro* following the manufacturer´s protocol.

For ELISA, NHA were seeded at 5.0 x 10^4^ cells/well in 6-well plates (Eppendorf) and incubated for 48 h with 5.0 x 10^9^ EVs/mL; H1 and H2 EVs in the presence or absence of miR-146a-5p inhibitor, H1 cells with WT or overexpression of NUMB in the presence of miR-146a-5p mimics, miR-146a-5p mimics combined with the Notch inhibitor DAPT, or DAPT during silencing of NUMB with a siRNA. Quantitative ELISAs for IL-6 (ab46042; Abcam), IL-8 (ab46032; Abcam), MCP-1 (ab179886; Abcam) and CXCL-1 (ab190805; Abcam) were performed to verify and quantify cytokine secretion according to the manufacturer´s instructions. Absorbance was measured using a Multiskan FC Microplate Photometer (Thermo Fisher). The experiments were performed in triplicate.

**2.14 Assessment of H1 growth cocultured with activation NHA conditioned media**

To determine the specific cytokine affecting growth of H1 cells by activated NHA cells on H1 cells, NHA were seeded at 5.0 x 10^3^ cells/well in 100 μL into a 96 well dish (Nunc) and left for 48 h. Then, NHA were incubated with 5.0 x 10^9^ EVs/mL from H1 or with PBS for 48 h. After 48 h, EV containing media was exchanged with fresh media for another 48 h. Conditioned media was then sterile filtered and transferred to a 96-well plate containing 5.0 x 10^3^ H1 cells/well. When transferring NHA-CM to H1 cells in a 96-well plate, 0.25μg/mL of either anti-CXCL1, anti-MCP-1, anti-IL-8 or anti-IL-6 (R&D Systems, Minneapolis, United States) antibodies were added. After 48 hours, proliferation of H1 cells was assessed by adding 10uL of the WST-1 cell proliferation reagent to each well and incubating for 3 h at 37^0^C. Absorbance of plates was measured at 450 nm using a Multiskan FC Microplate Photometer (Thermo Fisher Scientific).

**2.15 Microarray analysis of differentially expressed miRNAs in EVs**

To determine miRNAs differentially expressed in EVs, a microarray profiling was done on EVs collected from 3 MBM cell lines (H1, H2, H3) and from NHA and melanocytes in duplicates. Total RNA was isolated from samples using the RNeasy Micro Kit (Qiagen, Düsseldorf, Germany).

Affymetrix GeneChip® miRNA Array v4.0 st (Thermo Fisher Scientific) were performed using 500 ng of total RNA, with RIN of >9, according to the manufacturer’s user guide (FlashTag™ Biotin HSR RNA Labeling Kit For Affymetrix® GeneChip® miRNA Arrays P/N 703095 Rev3). Briefly, 500 ng of total RNA were subjected to a Poly (A) tailing with a PAP Enzyme, a biotin labeling was performed with a T4 DNA Ligase using the FlashTag^TM^ Biotin RNA Labeling kit (Thermo Fisher Scientific). Then, the labeling reaction was checked by an Elosa QC Assay before performing hybridization. GeneChip Eukaryotic Poly-A RNA Controls and Control Oligonucleotide B2 were added to the hybridization mix. 130 µl of the hybridization cocktail were injected onto the arrays. Arrays were incubated at 48^0^C and 60 rpm in a GeneChip^TM^ Hybridization oven (Thermo Fisher Scientific) for 16 h. Then arrays were stained and washed with the GeneChip® Hybridization Wash and Stain kit using the Fluidic script FS450_0002 (GeneChip® Expression Wash, Stain and Scan User Guide for Cartridge Arrays P/N 702731).

Raw CEL files of Affymetrix miRNA v4 microarrays were processed using a standard pipeline in Transcriptome Analysis Console (TAC v.4.0.1.36). Microarray data were preprocessed using the “RMA+DABG” algorithm on human probes. Only mature miRNAs, which show log_2_ expression above 3 in at least one sample were considered for further analysis in R/Bioconductor v.4.1.0, similar to previously described (1). Differentially expressed miRNAs were detected using *limma* R/Bioconductor package. Statistical significance was assigned based Benjamini-Hochberg’s false discovery rate (FDR). Volcano plot represents all considered miRNAs, the thresholds show FDR of 0.01 (horizontal) and log2FC of ±2 (vertical).

**2.16 RNA Protection Assay of EVs**

To evaluate the presence of miR-146a-5p internally in EVs, an RNA protection assay was performed. 5.0 x 10^9^ EVs were diluted in equal volumes PBS. Samples were incubated with 0.05mg/mL RNase A only, 0.05mg/mL RNase A + 1% Triton X, or equal volumes PBS control for 20 minutes at 37^0^C. RNA was extracted and qPCR quantification of miR-146a-5p was performed as described in Section 2.16.

**2.17 Real-time quantitative PCR (RT-qPCR) of miR-146a-5p and NUMB levels in EVs and cells**

Total RNA from EVs and cells were extracted using miRNeasy Tissue/Cells Advanced Kit (Qiagen). Quantification of RNA samples was done on the Nanodrop 1000 (Thermo Fisher Scientific). The relative quantifications of miRNAs of interest were performed by RT-qPCR. Extracted total RNA from EVs and cells underwent reverse transcriptase reaction using the miRCURY LNA RT Kit. 20 ng of RNA was used per reaction and spike in template RNA UniSp6 RNA/C. elegans cel-miR-39-3p (RNA Spike-In Kit, For RT; Qiagen) were added according to the manufacturer’s protocol. miRCURY LNA SYBR^®^ Green PCR Kit (Qiagen) was used for the PCR reaction. U6 or miR-103a-3p was used for normalization of cell RNA, but due to the instability of endogenous controls for EVs, C. elegans cel-miR-39-3p was used for normalization. ΔΔCt were calculated for relative quantification of expression.

Using the same protocol, relative NUMB expression levels normalized to U6, were analyzed in NHA cocultured with EVs derived from melanocytes, NHA or MBM cell lines H1, H2, and H3, or miR-146a-5p mimic. Downstream Notch, HES1, HEY1 and CCN1 were analyzed with the same method in NHA cells in the presence of H1-EVs, miR-146a-5p mimic, NUMB siRNA or NUMB overexpression. Primers used for the qPCR experiments are listed in Supplementary Table 1.

**2.18 In*-situ* hybridization (ISH) on patient MBM**

miRNAscope HD Assay Red (cat. no. 324500; Advanced Cell Diagnostics, La Jolla, CA, USA) was used to visualize miR-146a-5p in patient MBM tissue sections and *ex vivo* mouse brain tumors, according to the ACD formalin-fixed paraffin-embedded (FFPE) tissue protocol. Slides were imaged using an Olympus VS120 S6 Slide scanner (Olympus Life Science Solutions, Shinjuku, Tokyo, Japan).

For quantification ISH images were taken with TRITC filter as Fast Red probe is fluorescent under rhodamine filters. Ten equally sized representative areas were selected from each patient sample using QuPath 0.23 and exported to ImageJ v1.53. The mean gray value was quantified from each and divided by the area for a normalized value. Final values were determined by calculating the mean of all ten areas for each sample. Data are displayed as mean ± SD. Groups were compared using the Welch t-test.

**2.19 Analysis of miR-146a-5p levels in serum**

Patient serum samples were collected at the Dermatology Clinic in Dortmund (Prof. Dorothée Nashan, Dr. Sonja Dengler), at the University Clinic in Mannheim (Prof. Jochen Utikal, Klinische Kooperationseinheit Dermato-Onkologie, DKFZ) and at the University of Luxembourg. Ethical approval from the respective ethics committees in Germany and Luxembourg was obtained before sample acquisition. Written informed consent was received from all healthy controls and melanoma patients. Additional information on patient samples is given in Supplementary Table 2. Serum was aliquoted and frozen immediately after collection and stored at -80^0^C until further processing.

EVs were isolated from 250 µL of frozen patient serum using the Exoquick^TM^ Exosome Precipitation Solution (SBI, System Biosciences) according to the manufacturer’s instructions. Precipitated EVs were resuspended in 200 µL PBS. Size distribution profiles (nm) and concentration measurements (particles/mL) of isolated EVs were obtained using the NanoSight NS300 instrument (NanoSight Technology, Malvern, UK). Samples were diluted 500- to 2000-fold in PBS to obtain the ideal particle-per-frame value (20–60 particles/frame). Sample injection was performed with a syringe pump set at 40 µL/s speed at RT. The camera (sCMOS) was set with a gain of 73 and a shutter of 696 (camera level 10) and a gain of 146 and a shutter of 890 (camera level 11). For each analysis, 3 1-min videos were captured, representing 4494 frames, and analyzed with the nanoparticle tracking software NTA 3.3 Dev Build 3.3.301 with a detection threshold set at 3. The capture and analysis settings used were manually set according to the manufacturer’s instructions (Nanosight NS300 User manual MAN0541-02-EN).

Total RNA extraction from 200 µL isolated exosomes was performed using the miRNeasy serum/plasma kit (Qiagen) according to the manufacturer’s instructions. As an internal calibrator and to control for variations in recovery and amplification efficiency between RNA preparations, a mix of cel-39 and cel-54 exogenous controls (Qiagen) was spiked into the samples. RNA was eluted with 14 μL of RNase-free water. 4 µL out of 12 µL total exosome RNA was reverse transcribed using the miScript RT II kit (Qiagen) with HiSpec buffer specifically amplifying only mature miRNAs.

Due to generally low amounts of miRNAs extracted from exosomes, a miRNA pre-amplification step was included before performing qPCR. The 1:5 diluted cDNA was pre-amplified with the miScript PreAMP PCR kit (Qiagen) according to the manufacturer’s instructions, using specific primers: miRTC (internal Qiagen miRNA reverse transcription control), cel-54 and cel-39 (calibrators), miR-16-5p, miR-23a-3p, let7a, miR-223-3p and miR-451a (positive controls), and miR-146a-5p and miR-146b-5p. Quality controlled pre-amplified cDNA was diluted 1:20 and qPCR was carried out using specific 10X miScript primer assays (Qiagen) for individual miRNAs (mentioned above) and iTaq Universal SYBR Green Supermix (Biorad) on a CFX384 Detection System (Biorad). The spike-in controls and the target miRNAs were analyzed in parallel for each sample. Data were calibrated using cel-39 expression and normalized using global mean/miRNA (2).

**2.20 mRNA sequencing of NHA and differential expression analysis**

NHA were seeded at a concentration of 2 x 10^5^ cells/well in a 6-well plate. mRNA sequencing of NHA either exposed to miR-146a-5p mimic or miR-NC mimic (negative control) was performed by Novogene (Beijing, China) according to company protocols. Briefly, 1μg of RNA per sample was used to create sequencing libraries generated with NEBNext®UltraTMRNA Library Prep Kit for Illumina® (NEB, USA) following manufacturer’s recommendations. Sequencing was performed using 150 bases paired-end on an Illumina Novaseq sequencer with a targeted sequencing depth of 60 Million reads/samples. Differential expression analysis of NHA exposed to miR-146a-5p mimic or miR-NC was performed using the DESeq2 R package v1.16.1 (Bioconductor open-source software for bioinformatics). DESeq2 provide statistical routines for determining differential expression in digital gene expression data using a model based on the negative binomial distribution. The resulting P values were adjusted using the Benjamini and Hochbergh´s approach for controlling the false discovery rate (FDR). Genes with FDR-adjusted P-values < 0.05 found by DESeq 2 were assigned as differentially expressed.

**2.21 In silico analysis of potential binding partners to miR-146a-5p**

Three target prediction databases, TargetScan 7.1 (<http://www.targetscan.org/vert_71/>), miRDB (http://mirdb.org/), and microT-CDS (http://www.microrna.gr/microT-CDS) were used to determine predicted binding partners for miR-146a-5p. Each database provided a list of predicted mRNAs, which were combined with our own differential mRNA sequencing data to create a list of combined computed and experimental potential target genes of miR-146a-5p.

**2.22 Pulldown assay**

A miRNA pulldown assay was used to investigate the mRNA binding partners of miR-146a in NHA cells. Samples were sent to and processed by Shanghai Yunxu Biotechnology (Shanghai, China) according to their standard protocols. Briefly, biotinylated miR-146-5p and control miRNA were transfected into NHA cells and cell lysates were collected after 48 h. Lysates were incubated with M-280 streptavidin magnetic beads (Invitrogen) and 10 μL of yeast tRNA on a rotator at 4^0^C overnight. The bound miRNA/mRNA complexes were purified by adding 750 μL of TrIzol (Invitrogen) per sample and 250 μL of water to the input and the pull-down beads for further RT-qPCR analysis. 3 wells were analyzed for each sample. The primers used for pulldown are shown in Supplementary Table 3.

**2.23 Dual-luciferase reporter gene assays**

To verify the target relationship between miR-146a-5p and NUMB**,** NHA cells were transfected with a luciferase construct containing NUMB with the wild-type (WT) or a mutated version of the binding site and then co-cultured with miR-146a-5p mimic or a scrambled control (miR-NC) (GenePharma Co., Ltd, Shanghai, China). Luciferase activities were detected after 48 h of transfection by using a Dual-Luciferase Reporter Assay System (Promega Corporation, Madison, USA) according to the manufacturer’s instructions.

Briefly, NHA were grown at a concentration of 1.0 × 10^4^ cells/well in a 96-well dish. 75 μL Dual-Glo® Luciferase Assay Reagent was added to the wells and incubated at RT for 30 min. Firefly luminescence was then measured by an Ensight Multi Mode plate reader (PerkinElmer). Dual-Glo® Stop & Glo® Reagent was then added to each well and incubated for 30 min. Finally, Renilla luminescence was measured using the Ensight Multi Mode plate reader (PerkinElmer).

**2.24 Silencing and overexpression of NUMB in NHA**

siRNAs (siNC, siNUMB) and overexpression plasmid vectors (PCDNA3.1, vector containing NUMB or empty vector) were prepared by GenePharma (Shanghai, China). 2.0 x 10^5^ NHA cells/well were seeded in a 6-well plate overnight. The cells were then treated for 6 h, either with 6 μL siRNAS (20 μM of siNC or siNUMB) and 6 μL lipofectamine 2000 (Thermo Fisher), or overexpression plasmid vectors (1 μg of vector containing NUMB or empty vector) and 6 μL lipofectamine 2000 (Thermo Fisher). After 6 h, the growth medium was exchanged, and the NHA cells were cultured for another 48 h. The cells were then collected, and the proteins were extracted for WB according to standard protocols. siNUMB 5’ to 3’: GGUUAAGUACCUUGGCCAUTT. siNC 5’ to 3’: UUCUCCGAACGUGUCACG.

**2.25 Construction of a miR-146a-5p knockdown (KD) H1_DL2 cell line**

Mcherry-hsa-miR-146a-5p inhibitor sponge (5’ to 3’): TAACCCATGGAATTCAGTTCTCACGATAACCCATGGAATTCAGTTCTCAACCGGTAACCCATGGAATTCAGTTCTCATCACAACCCATGGAATTCAGTTCTCATTTTTTC) and mcherry-control lentiviruses (5’ to 3’: TTCTCCGAACGTGTCACGT) with a neomycin resistant region were synthesized by Genechem (Shanghai, China). For transfection, H1_DL2 cells were seeded in 6-well plates overnight and transfected using Lipofectamine 3000 (Invitrogen, CA, USA) according to the manufacturer’s instructions. Knockdown was confirmed with qPCR of miR-146a-5p levels in H1-DL2 cells normalized to U6 transcripts. Flow cytometry was used to sort mCherry-labeled, stably transfected cells. Cells were harvested for WB analysis 48 h after transfection, and the H1_DL2 KD cells were scaled up and frozen down for subsequent experiments.

**2.26 Studies of *in vivo* effects of miR-146a-5p knock-down in H1_DL2 cells**

Female nude mice were injected intracardially with 5.0 x 10^5^ MBM cells using ultrasound guidance, either H1_DL2 cells where miR-146a-5p was knocked down (H1_DL2 miR-146a-5p KD), or with H1_DL2 cells where a scrambled miRNA was used (H1_DL2 miR-NC KD; n = 10 mice in each group). The mice were imaged with BLI at weeks 4 and 6. The radiance values in regions of interest (ROIs) were registered in ROIs placed over the head and the body regions. Mean radiance values for head and body were calculated in GraphPad Prism v9.0 software (GraphPad Software Inc.). Animal survival was recorded, Kaplan-Meyer survival curves were plotted in GraphPad Prism v9.0 software (GraphPad Software Inc.), and a log-rank analysis was performed to determine the statistical significance of the differences in survival.

**2.27 *Ex* vivo immunohistochemical analysis of tumors from mice**

Paraffin-embedded mouse brain samples from either miR-146a-5p KD or deserpidine study were sectioned (4 μm) and mounted onto microscopic slides. For immunohistochemistry, deparaffinized sections were incubated with Ki67 (dilution 1:800; Cell Signaling Technology, Beverly, MA, USA) or GFAP (1:250; Abcam, Cambridge, UK) primary antibody at 4^O^C overnight, rinsed with PBS, and incubated with goat anti-rabbit secondary antibody (dilution 1:200; ZSGB Biotechnology, Beijing, China). One representative image from 5-6 mice in each group was taken using a Leica DMi8 microscope (Leica Microsystems). For each Ki67 image, positively stained nuclei were counted using QuPath v0.2.3 software’s cell classification (3). Positively Ki67-stained cells are presented as a percentage of a total number of cells counted.

For GFAP quantification whole slide images were acquired using an Olympus V120 slide scanner (Olympus Corporation, Tokyo, Japan). Using QuPath software a neural network pixel classifier was trained to separate the image into tumor, non-tumor tissue, cerebellum, and background/non-tissue. These preliminary classifications were then manually optimized. Tumor edge was defined as a 300 µm border around the tumors. Inside the different regions, all pixels with an intensity of DAB staining above a fixed threshold were considered positive for the antigen, and the total area of positive staining was quantified.

Statistical analysis of image analysis data was done using Rstudio software (RStudio 2022.07.1+554). Data are displayed as mean ± SEM. Groups were compared using the Welch t-test.

**2.28 3D structure modeling of miRNA and virtual screening of drug candidates**

The Homo sapiens RNA mir-146a-5p stem-loop sequence was acquired from miRBase database (http://www.mirbase.org) with accession ID of MI0000477. The sequence was submitted to MC-Fold/MC-Sym, a service for RNA secondary and tertiary structure prediction. MC-Fold outputs the secondary structures, and the hairpin loop of the pre-miRNA was subsequently submitted to MC-Sym to predict the 3D structure.

The dock module in Molecular Operating Environment (MOE v2015.1001) was used for structure-based virtual screening. The modeled 3D structure of mir-146a-5p were defined as receptor. The DrugBank database were defined as VS library for miR-146a-5p. Prior to docking, the force field of AMBER12: EHT and the implicit solvation model of Reaction Field (R-field) were selected. The orientation of the hydrogens was optimized by LigX module at pH = 7.0 and T = 300^0^K. For flexible docking, the docked poses were ranked by London dG scoring first, then a force field refinement was carried out on the top 10 poses followed by a rescoring of GBVI/WSA dG. After flexible docking, the ranked top 100 hits of each compound library were finally identified.

**2.29 Apoptosis study after *in vitro* deserpidine treatment**

To assess the effects of deserpidine on H1 cells, apoptosis was studied using a Dead Cell Apoptosis Kit with Annexin V for flow cytometry (Thermo Fisher Scientific), according to the manufacturer’s instructions. 1.5 x 10^6^ H1 cells were plated in 6-well plates and left for 24 h before treating with 0 μM (negative control), 10 µM, 20 µM or 30 µM deserpidine for 72 h. Then, cells were washed with cold PBS and trypsinized using 0.25% Trypsin/EDTA, collected and centrifuged at 1200 rpm for 5 min. Cells were washed with cold PBS and resuspended in 100μL 1X Annexin V binding buffer. 5μL of AlexaFluor®488 and 1μL of 100 μg/mL propidium iodide (PI) was added and cells were incubated for 15 min in the dark at room temperature. 400 μL of 1X Annexin-binding buffer was added after incubation, cells were transferred to ice and immediately analyzed using BD LSR Fortessa Flow cytometer (Becton Dickinson, NJ, USA). FITC-A (530 nm, Annexin V) and PE-A (>575 nm, PI) channels were used in a two-parameter histogram. FlowJo software v10.7.1 (Tree Star Inc., Ashland, OR, USA) was used to analyze raw data using the following cell populations: Q1: live cells (Annexin V−/PI−), Q2: early apoptosis (Annexin V+/PI−) Q3: late apoptosis (Annexin V+/PI+), Q4: necrosis (Annexin V−/PI+).

**2.30 *In vivo* drug studies**

First, an animal study was performed at the Shandong University, certified by the Association for Assessment and Accreditation of Laboratory Animal Care International*.* 6-8 weeks old female nude mice were anesthetized with 3% sevoflurane and maintained with 1.5% sevoflurane during the injection procedure. The mice were fixed in a supine position on a 37^0^C heating pad to maintain core temperature. 5.0 x 10^5^ cells in 0.1 mL sterile PBS were injected during 20 s into the left cardiac ventricle of each mouse using a 30G insulin syringes (Omnican50, B. Brain Medical AS, Vestskogen, Norway), by ultrasound guidance (Vevo 225 2100 Imaging System, VisualSonics Inc.). After tumor cell injection, the mice were divided into two groups (n = 10 mice in each group). One group received intraperitoneal (i.p.) injections of 0.5 mg/kg deserpidine in 100 μL solvent according to the manufacturer’s description, every 3^rd^ day for 7 weeks, the other group received 100 μL solvent i.p. every 3^rd^ day for 7 weeks. The mice were monitored daily after injection and BLI was carried out every week for 4 weeks to evaluate tumor burden. Animal survival was recorded and analyzed using GraphPad Prism v7 software (GraphPad Software, Inc.). The brains were harvested, fixed in 4% paraformaldehyde, and embedded in paraffin for subsequent analysis.

Next, the animal study was repeated at the University of Bergen, using MRI to study MBM tumor development in detail. 20 female NOD/SCID mice were injected i.c. with tumor cells with the procedure as described above and divided into 2 groups. 10 mice received i.p. injections of 0.15 mg/kg deserpidine in 100 μL solvent 3 days per week, while 10 mice received i.p. injections of 100 μL solvent 3 days per week. The mice were monitored daily after injection and MRI was carried out at weeks 4 and 6 to evaluate tumor burden. The brains were harvested, fixed in 4% paraformaldehyde, and embedded in paraffin for subsequent miR-146a-5p expression analysis.

**2.31 Statistical Analysis**

All statistical analyses including one and two way-ANOVAs, Student T-tests, IC_50_ calculations and Kaplan-Meier survival curves were generated using Prism 9 (Version 9.0.2 for Mac OS, GraphPad Software, San Diego, California USA). P < 0.05 was regarded as being statistically significant for all tests.

References

1. Nazarov PV, Reinsbach SE, Muller A, Nicot N, Philippidou D, Vallar L*, et al.* Interplay of microRNAs, transcription factors and target genes: linking dynamic expression changes to function. Nucleic Acids Res **2013**;41:2817-31

2. Margue C, Reinsbach S, Philippidou D, Beaume N, Walters C, Schneider JG*, et al.* Comparison of a healthy miRNome with melanoma patient miRNomes: Are microRNAs suitable serum biomarkers for cancer? Oncotarget **2015**;6:12110-27

3. Bankhead P, Loughrey MB, Fernández JA, Dombrowski Y, McArt DG, Dunne PD*, et al.* QuPath: Open source software for digital pathology image analysis. Scientific Reports **2017**;7:16878
